# Evaluating the resilience of residential buildings during a pandemic with a sustainable construction approach

**DOI:** 10.1016/j.heliyon.2024.e31006

**Published:** 2024-05-14

**Authors:** Ali Heydari, Hamidreza Abbasianjahromi

**Affiliations:** a Master of Engineering and Construction Management from Tehran azad University of Science and Research, Tehran, 1476656581, Iran; b Faculty of Civil Engineering, K. N. Toosi University of Technology, Tehran Province, Tehran, Mirdamad Blvd, No. 470, 19697-64499, Iran

**Keywords:** Residential building, Sustainability, Pandemic, COVID-19, Green building, MCDM, Home quarantine, Pandemic resistance

## Abstract

The COVID-19 pandemic has been a catastrophic event that has affected all aspects of human life worldwide. Due to the high genetic mutations of the virus, there has been a prolonged search for definitive therapeutic discovery, leading to extended periods of home quarantine. As a result, homes have become multipurpose spaces for work, education, sports, and other daily activities. Given the importance of residential buildings as the first line of defense against possible future pandemics, a model has been proposed to assess their readiness to handle pandemics using a sustainable development approach. This study investigates the most crucial criteria for evaluating residential buildings and applies them in a Multi-Criteria Decision Making (MCDM) process. The final evaluation model is presented using the SWARA and COCOSO methods, along with a set of criteria selected and weighted by experts. The study shows that the criteria related to health and safety are more critical than other sectors. Buildings that meet the standards of this group better are likely to have a higher score.

## Abbreviation list

AHP: Analytic Hierarchy Process
ANP: Analytic Network Process
ARAS: Additive Ratio Assessment
BIM: Building Information Modelling
BREEAM: Building Research Establishment Environmental Assessment Method
BWM: Best-Worst MCDM method
CASBEE: Leadership in Energy Assessment System for Built Environment Efficiency
COCOSO: A Combined Compromise Solution
COPRAS: Complex Ratio Assessment
CRITIC: Criteria Importance Through Intercriteria Correlation
DEMATEL: Decision-Making and Evaluation Laboratory
EDAS: Evaluation based on Distance from Average Solution
GBI: Green Building Index
GBCS: Green Building Certification Systems
LEED: Leadership in Energy and Environmental Design
MCDM: Multi-Criteria Decision Making
MEW: Multiplicative Exponential Weighting
SAW: Simple Additive Weighting
SDG: Sustainable Development Goals
SWARA: Step-wise Weight Assessment Ratio Analysis
TOPSIS: Technique for Order of Preference by Similarity to Ideal Solution
UVC: Ultraviolet-C
WASPAS: Weighted Aggregated Sum Product Assessment
WEF: World Economic Forum
WHO: World Health Organization
WPM: Weighted Product Model
WSM: Weighted Sum Model

## 1 Introduction

On December 31st, 2019 [1], a new virus called Severe Acute Respiratory Syndrome Coronavirus 2 [2], which was later named COVID-19 by the World Health Organization [3], was discovered in Wuhan city, China. Due to its unique characteristics and the lack of reliable treatments and vaccines for a long time, it not only posed a serious threat to human health on a global scale [4], but also caused unemployment and poverty [5], leading to a deeper impact on the global economy than the 2008–2009 global financial crisis [3]. During the COVID-19 pandemic, many countries began to impose strict transmission restrictions and home quarantine as the first solution to prevent the outbreak of the virus and reduce mortality rates [6]. As of April 7, 2020, about 3 billion people worldwide were quarantined to protect themselves from the virus and reduce its transmission, according to the World Economic Forum (WEF) [7], Despite having advanced healthcare systems, facilities, equipment, and medical knowledge, even developed countries faced despair and helplessness in the face of this catastrophe [8]. In such a situation, the priority is the safety of the majority of people who are locked up in their homes without the chance to go to hospitals that are out of empty beds [9].

The outbreak of the COVID-19 pandemic compelled a significant portion of the population to remain confined to their homes, transforming them into multifunctional spaces for work, education, exercise, relaxation, and other activities. Residential buildings, in turn, were tested for their ability to be resilient, adaptable, and compatible, in an effort to ensure the safety of their occupants without much prior planning. Consequently, people's interaction with different parts and spaces of their homes increased, leading to a higher consumption of resources such as electricity and water compared to pre-pandemic conditions. In addition to the prevailing health and hygiene emergency conditions, the pandemic and resulting home quarantine have made residential buildings the focal point of human societies [10]. It is, therefore, essential to prepare for potential future pandemics and lockdowns by upgrading the health and safety standards of residential buildings [11,12]. These structures are vital to preserving and sustaining communities, and their resilience and sustainability are critical to ensuring the safety and well-being of their occupants [13].

Thus, even after the cessation of the COVID-19 pandemic, it is imperative that homeowners make their houses more resistant to future pandemics and lockdowns by adopting greener and more sustainable practices. By doing so, they can ensure that their homes remain safe, comfortable, and functional in the face of adverse and unpredictable conditions.

In this scenario, it is essential that future residential buildings cater to human needs during pandemics by addressing three fundamental questions: (1) How can disease transmission be minimized? (2) How can negative environmental effects be reduced? (3) How can the quality of life of people spending most of their time at home be enhanced?

One of the keys to creating a healthier society, developing a stronger healthcare system, reducing poverty and inequality, achieving cleaner air, and creating a healthier environment is to move towards the Sustainable Development Goals (SDGs) [14], It is worth noting that the SARS-COV-2 virus outbreak has endangered the execution of the UN's Sustainable Development Goals by 2030 [15]. Therefore, following up and applying the SDGs is even more crucial in pandemic situations, as it can help to solve many problems related to the lack of water and food or poor health conditions. This, in turn, can transform a global threat into a global golden opportunity [16,17].

During the pandemic, residential buildings have become essential hubs of human society. It is important to consider the benefits of implementing SDGs on residential buildings to mitigate the effects of the pandemic. Therefore, it is necessary to evaluate residential buildings based on sustainable development criteria while taking into account pandemic conditions. The sustainability requirements of residential buildings currently in use should be reviewed to adapt to the current state of the COVID-19 pandemic and to prepare for future pandemic scenarios such as microbial terrorism or viral mutations [9].

This study aims to extract the most significant sustainability criteria for buildings and then use them to evaluate residential buildings' sustainability in pandemic conditions. Unlike other research and regulations that consider all sustainable construction criteria for building evaluation, this research focuses on identifying sustainable construction criteria that prevent residents from getting infected and the disease from spreading during their home quarantine. These criteria provide a healthy and high-quality environment for residents and have minimal negative effects on the environment [18]. The process of evaluating pandemic-resistant sustainable residential buildings is a Multi-Criteria Decision Making (MCDM) process that factors in economic, social, and environmental aspects of sustainable development as well as the characteristics of a building that maintain the health and comfort of residents during quarantine and prevent the spread of pathogens [19]. Due to the nature of this research and the studies performed, the integrated use of Step-wise Weight Assessment Ratio Analysis (SWARA) and Combined Compromise Solution (CoCoSo) methods to identify the most appropriate criteria for evaluating sustainable residential buildings resistant to pandemic conditions were approved. The selection of SWARA and CoCoSo for evaluating pandemic resilience in residential buildings within the sustainable development framework stems from their adaptability to address uncertainty and complexity inherent in such contexts. SWARA method, offers a straightforward approach for determining criteria weights, making it accessible to diverse stakeholders with varying expertise levels. This simplicity is especially valuable in situations where predefined priorities guide decision-making processes, as it streamlines the weighting process based on expert judgments. Meanwhile, The CoCoSo method, provides a flexible and stable method for multi-criteria decision-making, accommodating proportional assessments and dynamic decision contexts crucial for navigating the multifaceted challenges posed by pandemics. Traditional methodologies like the Analytic Hierarchy Process (AHP) and the Analytic Network Process (ANP), though valuable, may struggle to address the uncertainties and complexities inherent in pandemic environments. The emergence of SWARA and CoCoSo reflects their potential to encapsulate the multifaceted dimensions of sustainability amidst global health crises like COVID-19. Their adaptability and reliability underscore their capacity to confront pandemic resilience challenges within sustainable development frameworks, offering robust decision-making frameworks for assessing residential building resilience in uncertain and dynamic environments [[20], [21], [22]].

This study is impressive for its unique approach in evaluating the sustainability of residential buildings during the pandemic. By focusing on criteria that prevent infection transmission and ensure residents' well-being during home quarantine, this research makes a significant contribution to the field of sustainable development. Traditional evaluations often overlook the challenges posed by pandemics, but this study addresses these gaps by identifying and prioritizing criteria tailored to pandemic resilience. The use of both SWARA and CoCoSo methods represents a novel methodological approach, offering a comprehensive framework for assessing pandemic resilience within the sustainable development paradigm. This innovative combination of methodologies enhances the reliability and robustness of the evaluation process, enabling stakeholders to make informed decisions in uncertain and dynamic environments. By pioneering this innovative approach, this study lays the foundation for future research and policy initiatives aimed at enhancing residential building resilience in the face of global health crises.

This article will delve deeper into the background literature, methodology, discussion, and conclusion of the study. The background literature section will provide a comprehensive review of existing research on sustainable building practices, pandemic resilience, and multi-criteria decision-making methodologies. The methodology section will outline the research design, data collection methods, and analytical procedures employed in evaluating pandemic-resistant sustainable residential buildings, paying special attention to the integration of SWARA and CoCoSo methods. The discussion section will present the findings, analyze key insights, and explore their implications for theory, practice, and policy. Finally, the conclusion section will summarize the main findings and propose avenues for future research and action.

## 2 Background literature

To develop a comprehensive model for evaluating residential buildings in pandemic scenarios, emphasis is placed on sustainable development. Various aspects are explored, beginning with the examination of risks and consequences associated with previous pandemics. Subsequently, an assessment is made regarding how these risks impact residential structures, which serve as critical havens during such crises. Additionally, criteria aimed at mitigating disease transmission, fostering healthy living environments, and minimizing environmental harm are compiled and analyzed. A fundamental aspect involves the crafting of a MCDM framework tailored to the study's objectives. Therefore, an exhaustive review of literature across diverse domains, including pandemic studies from the COVID-19 era, experiences of home quarantine, sustainable building standards, SDGs, and MCDM models, is undertaken. Insights gleaned from prior research in these specialized realms are presented in this section.

### 2.1 Pandemic and home lockdown

In the wake of global pandemics such as COVID-19, residences have assumed multifaceted roles beyond mere dwellings. They became spaces where individuals could work, study, exercise, and seek solace amid enforced lockdowns. The advent of COVID-19 necessitated stringent measures, confining populations within their homes to curb viral transmission. Consequently, the pandemic underscored the need to reevaluate architectural paradigms concerning residential structures. Architects and urban planners confronted the imperative of reimagining home designs to safeguard occupants against infectious diseases. COVID-19 has underscored the significance of fostering healthy and secure residential environments, merging architectural considerations with public health imperatives.

Since taking refuge to the physical and built environment is one of the first solutions when a pandemic emerges and before developing medications for it, urban planning and architecture will no longer be the same as in the past. Under such circumstances, it is necessary to provide a virus-resistant built-environment that utilizes a multi-layered defense system. Based on the experiences gained from COVID-19 Pandemic, architects, planners and managers need to change current built environment designs to come up with measures to prevent the spread of the virus and create a healthier and safer environment; this new model should be able to provide new and practical tools and instructions [23].

In Wang's [24] article considering restricted activities of humans during pandemics in houses and indoors, buildings are known as places with the highest population density which are considerable in prevention and control the spread of a pandemic. Prohibition and control measures related to the society and buildings have become one of the most efficient solutions in the pandemic era.

China Assessment Standard for Green Building (GB/T 50378-2019) briefed the beneficial effects of a building in the prevention and control of COVID-19 into five aspects.

• Providing the essential practices to prevent and control pandemic.
• Provisioning facilities for controlling the pandemic.
• Decrease the likelihood of infection transmission and preventing cross infection.
• Preserving and boosting health condition of residents.
• Considering a healthy, safe and sustainable environment for residents to live and work during home quarantine.

In order to prevent and control pandemics effectively, it's important to consider how buildings can impact the health of their residents and their interactions with one another. As a result, it's necessary to review and revise the rules and guidelines for constructing green buildings in preparation for future pandemics [24]. A related article suggests that residential buildings built post-COVID-19 should incorporate lessons learned from the pandemic era. In addition to providing all the necessary facilities for living under normal conditions, these buildings should also come equipped with additional facilities to deal with possible future pandemics. This includes protecting residents from external environmental damages in case of infection, providing suitable conditions for other residents to avoid infection, and facilitating faster recovery for those who are infected [25]. It's crucial to acknowledge that the COVID-19 quarantine period had a profound impact on both physical and mental health. The prolonged period of social isolation and home quarantine had negative psychological effects on individuals. As a result, it's imperative to address this issue and prioritize the significance of residential buildings by providing a comfortable, healthy, safe, and sustainable environment for residents [26].

Tokazhanov et al. [9] also articulate the profound and extensive impact of the COVID-19 pandemic on a global scale. The necessity to uphold social distancing measures and curb the transmission of the virus precipitated widespread implementation of stringent home quarantine measures worldwide. Consequently, this paradigm shift transformed the role of residential buildings from mere shelters into multifunctional spaces accommodating work, exercise, education, leisure, and recreational activities. In reflection, the examination of pandemics and their impact on residential settings underscores a pivotal juncture in architectural discourse. The COVID-19 pandemic, in particular, precipitated a paradigm shift, compelling architects and urban planners to reassess the design and functionality of residential spaces. As homes assumed a central role in mitigating viral transmission, the need for adaptable and resilient built environments became increasingly apparent. Lessons gleaned from the pandemic era underscore the imperative of integrating health-conscious design elements into residential structures. This imperative extends beyond physical health considerations to encompass the psychological well-being of occupants during periods of prolonged isolation. Moreover, the pandemic served as a catalyst for revisiting sustainability standards within the construction industry. Acknowledging the inherent interdependence between residential sustainability and broader societal goals, there arises a pressing need to align construction practices with SDGs. The post-pandemic era demands innovative strategies and tools to cultivate sustainable, resilient, and socially conscious residential environments. Transitioning from crisis response to proactive design necessitates a reevaluation of existing sustainability assessment frameworks. The quest for sustainable residential solutions in a post-pandemic world mandates a holistic approach that prioritizes environmental stewardship, social equity, and public health. As we navigate the uncertainties of the future, the convergence of sustainability and pandemic preparedness emerges as a linchpin in shaping the built environment of tomorrow.

### 2.2 Sustainable buildings and sustainability rating tools

The pursuit of sustainable architecture and the development of rating systems for sustainability are pivotal steps in the evolution of architectural discourse. In the face of global challenges like the COVID-19 pandemic, it is paramount to reinforce residential structures with sustainable principles. As the construction industry takes on a greater role in advancing SDGs, the movement towards sustainable buildings becomes even more crucial. While progress toward sustainability targets may have been slow in the past, the pandemic has underscored the need to align construction practices with environmental stewardship, social equity, and public health. The growing demand for eco-conscious design and construction methodology is a reflection of a shift towards sustainable living. To achieve this, it is necessary to have robust frameworks for sustainability assessment that can measure the environmental, social, and economic impact of residential structures. By focusing on sustainable buildings and rating systems for sustainability, the construction industry can be guided toward a future characterized by resilience, responsibility, and environmental harmony. The construction industry plays a crucial role in advancing towards the SDGs. It is one of the largest industries in terms of consumption and depletion of natural resources and has extensive communication and interaction with other industries and society [27]. However, before the COVID-19 pandemic, progress towards achieving the SDGs was slow, the pandemic has made it worse, but it is a misconception to conclude that the SDGs are less important, applicable, and effective in these circumstances. In fact, further progress towards many SDGs could reduce the likelihood of the emergence of pandemics. In this case, all the countries around the world were dealing with the COVID-19 disease with a stronger health system, fewer people in poverty, and a healthier and safer environment [14,17]. In addition, making progress towards the targets of SDG 3 (good health and well-being) would clearly improve the condition of the health system under pandemic pressure [28]. Therefore, mankind can consider such critical situations as an opportunity for significant change [17,29]. Finally, To cope with possible future pandemic conditions, it is necessary to revise and improve the sustainability regulations of residential buildings to be more compatible and resilient [27].

Current sustainability assessment tools that are primarily in favor of environmental impact and energy performance of buildings under the impression of COVID-19 pandemic conditions must have a special approach to social and health aspects [9]. The COVID-19 outbreak changed the way people use their houses to a multitask place for resting, working, studying, and almost all the other daily routines. Hence, it is necessary to achieve all the goals of an ideal life that provides all human needs for work and life together with comfort, health, and well-being. Sustainable construction is one of the ways to achieve this purpose and because the construction sector causes up to 40 % of global carbon dioxide emissions and produces more than 0.5 billion tons of waste annually, as a result, it can be a threat to the SDGs, therefore in the post-COVID world, new tools and strategies are needed to achieve constructions with slight emissions, inexpensive operation and most importantly, living safely, healthily and comfortably for a long time. So, sustainable houses are more desired than ever and now that everything is returning to normal, gained experiences during the pandemic can be used to initiate forming a built environment with a high level of responsibility in the framework of sustainable construction that is optimized for pandemic conditions. Since the COVID-19 pandemic is not the first one and it is not supposed to be the last one, only the exact time of the next catastrophe is unknown [30].

The method presented in Tokazhanov, G. et al. [27] paper aims to evaluate the sustainability of residential buildings in pandemic conditions. Current sustainability assessment methods often focus on the three main pillars of sustainable development, namely environmental, economic, and social, with the COVID-19 pandemic emergence, people around the world have been forced into long-term home quarantine, underscoring the importance of making changes to the design of residential buildings to fit in and out of such situations. These changes require the use of sustainable building assessment tools in pandemic conditions, and recent research shows that current assessment tools cannot meet these needs. It is expected that the measures proposed in this study will help to address these needs for the development of pandemic residential buildings. Another article by Aidana et al. [31] states that the process of evaluating the sustainability of buildings is an evolving process full of challenges that are constantly changing in the direction of improving, and meeting current needs. Building sustainability is usually assessed based on economic, social, and environmental aspects of sustainable development. Many sustainability evaluating tools have been developed worldwide. The most important of which are BREEAM (Building Research Establishment Environmental Assessment Method), LEED (Leadership in Energy and Environmental Design), and CASBEE (Leadership in Energy Assessment System for Built Environment Efficiency) [32], building sustainability assessment tools include several sets and some indicators that determine sustainability within a frame [33,34]. Through these indicators, it is possible to understand the coverage of each tool in different areas of sustainability. For example, the WELL Regulation generally focuses on aspects of social comfort, and the LEED focuses mostly on energy-saving items [35]. Therefore, according to the experiences gained during the period of COVID-19 pandemic, the need for a tool to evaluate sustainable buildings that pay more attention to indicators related to the health of residents in pandemic conditions is more than before. Therefore, this study examines the opinions of various experts about weighting the criteria for evaluating sustainable buildings and prioritizing pandemic conditions and it shows that to overcome the current situation and prevent the recurrence of disasters such as the COVID-19 pandemic, new frameworks and tools are needed for sustainable assessment of residential buildings with a more effective response to pandemic conditions [26,30,36,37].

Aidana et al. conducted research on the green certification systems of residential buildings, taking into consideration the pandemic conditions. The study aimed to determine if the Green Building Certification Systems (GBCS) are equipped to properly assess the sustainability of residential buildings in the post-COVID era. The researchers formed specialized roundtables to provide a set of building sustainability criteria appropriate to pandemic conditions. Then, they assessed the readiness of building sustainability assessment tools (such as BREEAM, LEED, WELL, CASBEE) to the selected criteria. The study revealed that none of the GBCS fully meets the criteria for the stability of residential buildings appropriate to the pandemic period. Each GBCS focuses more on one of the sustainable development pillars. For instance, the WELL rating system covers health and safety well, while the LEED rating system is more prepared for environmental performance. BREEAM and CASBEE systems offer a more balanced approach, addressing all three sets of residential building sustainability criteria in pandemic conditions, namely health and safety, environmental resource consumption, and comfort, with a greater focus on occupant comfort. However, none of the GBCS are prepared to meet the criteria related to waste and wastewater management. Therefore, it is necessary to adapt the systems to new changes and standards for possible future pandemic conditions and evolving pandemics [35].

The World Health Organization [3] has also pointed out that many new buildings today do not provide adequate and standard conditions for their occupants [38]. Habitats with poor interior environment design can easily transmit pollution inside, leading to the spread of diseases. This can cause direct treatment costs and reduce the productivity of people due to their illness [39].

In summary, the COVID-19 pandemic has revealed critical deficiencies in residential buildings, necessitating enhancements in health and safety, resource consumption, and comfort. Through research by Tokazhanov and others [27,35], insights into touchless technologies and mental well-being optimization have emerged, signaling a push for pandemic-resilient construction.

Transitioning, the discourse on sustainable buildings and rating tools lays the groundwork for addressing pandemic-specific criteria in construction practices. Scholars advocate for holistic frameworks prioritizing health, safety, and environmental stewardship in pandemics. Building on pandemic-era insights, the next section explores sustainable building criteria during pandemics. From confined spaces to ventilation optimization, these criteria mitigate infection risks and address construction sustainability and occupants' health and comfort. The future of residential buildings lies in their adaptability and promotion of community well-being amid uncertain times like pandemic situations.

### 2.3 Sustainable building criteria during pandemic

The imperative of ensuring resilient and sustainable residential structures amidst pandemics has underscored the need for comprehensive frameworks and criteria. As the world grapples with the profound impact of the COVID-19 pandemic, attention shifts towards redefining the parameters of sustainable building practices. Amidst the backdrop of lockdowns and home quarantines, the vulnerabilities and shortcomings of existing residential buildings have become glaringly apparent. In response, scholars and practitioners advocate for integrating pandemic-specific criteria into sustainable construction practices. This section delves into the evolving discourse surrounding sustainable building criteria during pandemics, exploring innovative approaches aimed at fostering health, safety, resource efficiency, and community well-being in residential environments. During the lockdown, many deficiencies in residential buildings regarding health and safety hazards, consumption of environmental resources, and lack of personal comfort have been revealed. Tokazhanov's paper [27], suggests that improving safety and health conditions in residential buildings can be achieved through measures such as increasing the use of touchless technologies, deploying finishing and coating materials that can affect viruses viability, implementing smart technologies, expanding green sections in the house, enhancing comfort, improving communication technologies for better remote services, and paying attention to air quality, humidity, light, and temperature criteria [9]. In light of the COVID-19 pandemic and the possibility of future pandemics, Daniela [40], in a separate article, emphasizes the need to leverage the potential of residential dwellings. She suggests that integrating health and well-being elements in new constructions or existing structures can provide a robust response to the challenges posed by pandemics. Daniela advocates for the following key considerations to create residences that are healthy, safe, sustainable, and resilient in adverse conditions: 1. Utilizing green factors and spaces with easy access and a view of them. 2. Considering capabilities of pliability, openness to change, population density in spaces, and compatibility of building functions. 3. Reviewing and modifying the fundamental principles of sustainable construction and architecture, interior air quality, and thermal welfare. 4. Paying attention to wastewater management and health and water quality. 5. Proper waste management. 6. Choosing suitable finishing and consumable materials in interior design [40].

In one study, Yadegaridehkordi et al. [19] used fuzzy DEMENTEL to evaluate the sustainability of Green Building Index (GBI) using 6 primary criteria, 17 sub-criteria, and 54 associated sustainability indicators. The results showed that energy efficiency and environmental quality were the most important criteria. In the energy efficiency dimension, 'Design & Performance' was the most significant sub-criterion, while in environmental quality, 'Lighting, visual & acoustic comfort' was the most significant factor. The least important criteria were water efficiency and innovation. The study aimed to provide a model for evaluating sustainable residential buildings, considering pandemic conditions, and using MCDM methods to evaluate the desired structure.

In a related study [35] the author delineates the objective of augmenting existing sustainability assessment frameworks for residential buildings in the context of pandemics. The study endeavors to enhance established sustainability assessment systems by scrutinizing their evaluation criteria and proposing measures tailored to pandemic conditions. Moreover, the research highlights the inadequacy of current sustainability assessment systems, which predominantly focus on environmental, economic, and social aspects, in addressing the exigencies of pandemics. Drawing from the extant literature, the study gathers criteria pertinent to building sustainability during pandemics. Subsequently, through a roundtable method involving stakeholders and experts, the study scrutinizes, classifies, and compares these criteria to refine sustainability assessment tools. The criteria are divided into three main groups: health and safety, environmental resource consumption, and comfort. Each of these groups includes sub-categories and specific criteria. For instance, the health and safety group has sub-categories like PVP (Prevention of Virus Propagation), Mental Health, Air Quality, Water Quality, and Wastewater Management. The PVP sub-category includes criteria such as PVP1: Deployment of Intelligent New Technologies, PVP2: Touchless Technologies, PVP3: Self-Cleaning Surfaces and Spaces, PVP4: Suitable Interior Materials, PVP5: Natural Light, PVP6: Temperature and Humidity Control, etc. Similarly, other criteria are placed in their respective categories. All criteria are scored quantitatively on a 5-point scale. This study also compares several popular green building certification systems (WELL, LEED, BREEAM, CASBEE) in terms of their selected criteria and scores. However, the study found that these certification systems fail to cover important criteria for resistance in pandemic conditions, and therefore, they do not meet the needs of making changes in the design of green buildings to adapt to pandemic conditions and tolerate such conditions. Thus, it is clear that there is an urgent need to use proper tools for evaluating the sustainability of residential structures in pandemic conditions [27,35].

Therefore, it is necessary to solve the problem by using the experiences gained from the COVID-19 pandemic and providing new systems and tools that use appropriate and optimized criteria to evaluate residential buildings' sustainability in pandemic situations.

Ling Xin Yong's review [41] explores antimicrobial technologies for built environment surfaces, emphasizing ceramics, textiles, and metals like TiO2 and metal nanoparticles. Integration criteria include ease of application, effectiveness, and aesthetics. High-interaction indoor spaces like homes and schools, especially in urban areas with dense public transportation, benefit from antimicrobial surfaces. Challenges include varied built environment types and dynamic indoor conditions, requiring meticulous planning. Comprehensive evaluations are essential for understanding real-time impacts and fostering adoption across diverse environments. Another study by Melika Amirzadeh et al. [42] emphasizes the importance of architects and urban planners in enhancing urban resilience against pandemics, focusing on pandemic-resilient homes. Key criteria for pandemic-resilient homes include multifamily housing with private natural environments, touchless technological equipment, antibacterial surfaces, proper ventilation, and adaptive interior layouts. These criteria ensure safe living environments that minimize virus transmission and promote residents' well-being. Implementing such criteria across various spatial scales is essential for creating resilient urban environments against pandemics and other hazards. Future research should further develop and implement these criteria to improve urban resilience worldwide. The critical review by Mugahed Amran [43] emphasizes design approaches like sunlight and natural ventilation in mitigating infection risks in buildings. It advocates multidisciplinary efforts and recommends integrating public health training for engineers and architects. Emphasizing natural ventilation, improved filtration, and sunlight integration in building designs can enhance indoor air quality, curb viral infections, and promote ecological sustainability and energy conservation. This highlights the pivotal role of architectural and engineering strategies in combating the spread of infectious diseases like COVID-19.

Research conducted by Galym Tokazhanov, Aidana Tleuken et al. highlights that the COVID-19 pandemic and subsequent home-quarantine on a global scale have made the characteristics of residential buildings more important. The study evaluates new sustainability indicators across three categories: safety and health, consumption of natural and environmental resources, and comfort, with a focus on making buildings more resilient during pandemics. The opinions of experts were analyzed to identify the most important indicators, with the health and safety sector being deemed the most critical. Sub-categories of the health and safety sector include virus prevention, mental health, and air quality of the building. For example, the use of new intelligent and non-touch technologies has been identified as the most important criterion for preventing virus propagation. Additionally, providing outdoor open spaces and safe places for communities in quarantine to maintain mental health of residents has been recognized as a priority. Criteria for air filtration and separation of medical and infected people waste have also been introduced as important indicators for pandemic residential buildings [26]. Another research examines the impact of the COVID-19 pandemic on new residential buildings from an architectural perspective using space-building criteria. Criteria introduced in this research for new or retrofitting buildings include installation of confined spaces, separation of entrances and exits, and separation of interior spaces equipped with independent air conditioning systems with additional filters. These measures greatly reduce the possibility of infection and if residents become infected, by providing a suitable space while maintaining comfort, they prevent the virus from spreading further. However, implementing and applying these criteria in buildings can include costs that cause socio-economic disparities and ultimately health issues in society. As in the case of the COVID-19 pandemic, people with the ability to work remotely from home [44], provide home-based education for their children [45], utilize private vehicles rather than relying on public transportation [46], reside in detached houses as opposed to densely populated public housing complexes, and afford home delivery services instead of engaging in in-person shopping, have been able to mitigate the risk of infection to a greater degree than their socioeconomically disadvantaged counterparts. A similar socioeconomic disparity may also manifest in the realm of tailored housing solutions amid pandemics. To ameliorate this gap, designers and stakeholders must strategically leverage the most cost-effective emerging criteria to mitigate public health repercussions [25]. Therefore, experiences gained from the COVID-19 pandemic are expected to influence the development of residential buildings in the future as multi-purpose places. For example, distance work, education, or sports will be more important [47], just as previous pandemics have influenced the built environment, prompting increased focus on expanding green spaces, reforming sewer systems to combat diseases like cholera and plague, incorporating natural sunlight in residential units to inhibit the spread of tuberculosis, and enhancing ventilation systems following the outbreak of SARS-CoV-1 [48].

In conclusion, the discourse on sustainable building criteria during pandemics highlights the necessity of integrating health, safety, and resource efficiency considerations into construction practices. The research underscores the limitations of existing assessment tools in addressing pandemic-related challenges and emphasizes the need for optimized frameworks tailored to these exigencies. Through methodologies like fuzzy MCDM, AHP, and COPRAS, scholars have sought to refine existing evaluation systems and prioritize criteria conducive to pandemic resilience. However, the evolving nature of pandemics necessitates ongoing research and adaptation of assessment methodologies to address emerging challenges effectively.

Transitioning to the next section, the discourse on sustainable building assessment tools and decision-making approaches builds upon the foundation laid in evaluating criteria for pandemic-resistant residential structures. As the discussion expands to encompass diverse methodologies like SWARA, ANP, and CoCoSo, the focus shifts towards comprehensively evaluating complex systems and adapting decision-making frameworks to dynamic and uncertain environments. By exploring various assessment tools and methodologies, the subsequent section seeks to elucidate how these approaches contribute to fostering pandemic resilience and sustainability in residential buildings. Through a nuanced examination of common criteria and decision-making processes, the ensuing discourse aims to inform practitioners and policymakers alike in shaping the future of sustainable construction practices amidst the backdrop of global pandemics.

### 2.4 Sustainable buildings assessment tools and decision-making approach

In the realm of sustainable construction, the urgency to develop resilient building practices amidst global pandemics like COVID-19 has become increasingly evident. One of the critical challenges in navigating these unprecedented times has been the absence of a tailored tool for evaluating building sustainability criteria in light of pandemic resistance. While numerous scholarly inquiries have delved into sustainability indices within building regulations, the emphasis on pandemic exigencies has been relatively understated. Among notable studies, the work of Yadegaridehkordi et al. and Abdel-Basset et al. has shed light on methodologies such as GBI and AHP in evaluating sustainable construction. Despite these efforts, the deficiency in adapting traditional assessment tools to uncertain environments, particularly during pandemics, remains palpable. As the discourse intensifies around the need for comprehensive evaluation frameworks, the spotlight turns to subtle yet potent methodologies like SWARA and CoCoSo. Their simplicity, transparency, and capacity to capture the relative importance of criteria without relying excessively on pairwise comparisons make them promising candidates for assessing pandemic resilience in sustainable buildings. In this section, the suitability of SWARA and CoCoSo methods are explored within the context of pandemic resilience, shedding light on their efficacy in addressing the multifaceted challenges posed by global health crises. Based on the research and studies, it seems that the lack of an optimized tool to assess building sustainability criteria considering resistance to pandemic conditions was one of the main obstacles to crossing the pandemic situation as quickly as possible and getting ready to face future pandemics. Numerous scholarly inquiries have explored the assessment and prioritization of sustainability indices within the domain of building regulations, irrespective of pandemic exigencies. Noteworthy among these studies is the work of Yadegaridehkordi et al. [49,50], whose investigation focuses on GBI, a preeminent framework for evaluating sustainable construction in Malaysia. The study employs the fuzzy Decision-Making and Evaluation Laboratory (DEMATEL) approach to refine the GBI framework, which involves the discernment and hierarchical arrangement of sustainability metrics relevant to Malaysian green building endeavors. Sustainability evaluation is a complex process that takes into account economic, social, and environmental considerations [[51], [52], [53], [54]]. MCDM is the predominant methodological approach used for discerning and prioritizing the associated criteria and sub-criteria within environmental science scholarship [49,[53], [54], [55], [56]]. Another study by Abdel-Basset et al. [57] evaluates building sustainability in developing countries, emphasizing water management, energy efficiency, and environmental sustainability [51,54]. The AHP is a popular MCDM method used for decision-making [58,59]. but its inefficiency in uncertain environments led to its adaptation in a neutrosophic environment in this study [57]. The study's results indicate that, in developing countries, water efficiency is the most critical criterion, while energy efficiency is the least important criterion, regardless of pandemic conditions [39].

A research study by Raimondas Bliudžius et al. [60] emphasizes the importance of sustainability evaluation and monitoring within the context of global dynamics and developmental paradigms. The study suggests that MCDM methodologies should be used as a paramount approach for appraising sustainability, particularly within the domain of urban building management, which is characterized by a myriad of diverse criteria. The study employs the AHP to ascertain the relative importance of each criterion, and then devises a methodological framework to refine the evaluation of each criterion's significance. In the final analytical phase, the study uses two MCDM techniques, namely Complex Ratio Assessment (COPRAS) and Weighted Aggregated Sum the Product Assessment (WASPAS), to model the decision-making process comprehensively. By embracing this methodological framework, the study contends that a thorough assessment of property facets becomes feasible, thereby fostering optimal management practices and facilitating the development of more resource-efficient structures. Consequently, such endeavors are expected to improve residents' needs satisfaction, reduce energy consumption, and mitigate environmental pollution. In another study [61], the inefficiency of current sustainability frameworks and tools has been examined from another perspective. It is stated that despite the growing global importance of sustainable development and the environment, real estate projects are often considered only from a risk and efficiency perspective. In the limited cases that indicators and frameworks of sustainable development are used, only the three well-known dimensions of sustainability, namely the environment, economy, and society, are considered. This study suggests that a fourth dimension, namely the technological dimension, should be added to the process for assessing the sustainability of real estate projects. After studying the previous standards and literature, the study extracts the criteria and selects and rates the most relevant ones via a survey. Then, using MCDM methods, it presents a new proposed evaluation system. There are many resources available that evaluate the sustainability of residential buildings from different perspectives and using various methods of MCDM. However, due to the pandemic and the prevalence of COVID-19, there is now a need to evaluate residential buildings from the perspective of their resistance to pandemic conditions while still maintaining the principles of sustainable development.

At the time of writing, there were limited resources available to review and rank the evaluation criteria of pandemic-resistant sustainable residential buildings using MCDM methods. This is because of the novelty of this issue. Nevertheless, there are various building sustainability evaluation standards such as BREEM, LEED or GSAS that are used for evaluating and rating green buildings by using typical topics, such as water and energy, site consideration, substance input, and indoor and outdoor environments. These are regular assortment of residential buildings sustainability evaluating tools [62,63]. However, these residential buildings sustainability standards are inefficient in solving a wide range of problems that arose during the emergence of COVID-19 disease, such as domestic violence, energy, waste, water management, and food supply. Thus, it is vital to be more attentive to the environmental performance aspects. Therefore, there is a necessity to review common sustainability systems and their requirements.

In a study by Galym Tokazhanov et al. [64], the sustainability criteria of pandemic-resistant residential buildings were evaluated in three sections: Safety and health, Consumption of Natural and Environmental Resources, and Comfort. The opinions of various experts and stakeholders were analyzed to weigh the pandemic-resistant sustainability indicators using the Dotmocracy method. This is a collaborative decision-making technique that allows stakeholders and experts to vote on priorities and rank desired items in order of priority from highest to lowest. This study also points out that building sustainability assessment tools can vary by country and geographic area [65]. For instance, water resources management and water quality are more important for countries of Central Asia than for European countries or the United States [66]. After analyzing the final votes of the stakeholders and analyzing the data, it was concluded that the health and safety section has higher importance compared to other sectors. The criteria for preventing the spread of the virus, residents' psychic health, and indoor air quality were selected as the most important subsets of the health and safety sections [26]. In another paper by Saeed Kamranfar [67], the discussion revolves around analyzing green construction development barriers using a hybrid decision-making method based on DEMATEL and ANP. This approach integrates DEMATEL's capacity to identify causal relationships among factors and ANP's capability to model interdependencies in complex decision networks. Through paired comparisons, experts evaluate the relationships and influences among different barriers such as economic, managerial, social, cultural, and knowledge-related obstacles to green construction development. This method enables a comprehensive understanding of the interplay between various factors affecting the implementation of green buildings, providing insights into effective strategies to overcome these barriers and promote sustainable construction practices. Moreover, the study underscores the importance of considering specific context, climate, and regional characteristics in devising strategies for green construction development, highlighting the significance of tailored approaches to address local challenges and foster sustainability in construction projects. Additionally, the paper contributes to methodological advancements by proposing modifications to the traditional DEMATEL-ANP approach, streamlining the questionnaire process, and enhancing the model's accuracy and efficiency, thereby improving its applicability in practical decision-making contexts within the construction industry.

Reviewed literature and resources highlight the significance of sustainability assessment in building regulations, encompassing various methodologies such as MCDM and the AHP. Notably, studies by Yadegaridehkordi et al., Abdel-Basset et al., and Raimondas Bliudžius et al. underscore the importance of criterion prioritization and comprehensive decision-making in evaluating sustainability. Despite emerging challenges like the COVID-19 pandemic, diverse assessment standards like BREEM, LEED, and GSAS continue to shape sustainability evaluations, with stakeholders prioritizing pandemic-resistant indicators such as safety, health, and resource consumption. The table below summarizes commonly assessed criteria, MCDM methods, and their application discussed in this section.

MCDM methods play a crucial role in evaluating complex systems with multiple conflicting criteria. Several MCDM techniques, including SWARA, AHP, ANP, and CoCoSo, have been utilized in various studies to assess the sustainability of residential buildings, especially in pandemic conditions. Each method has its strengths and weaknesses, influencing its suitability for different research contexts.

AHP provides a structured approach to decision-making by decomposing complex problems into hierarchies, making it easy to understand and implement. It allows for pairwise comparisons of criteria and alternatives, facilitating the determination of priorities. However, relies heavily on subjective judgments, and inconsistencies in pairwise comparisons can lead to biased results. It may also struggle with complex decision scenarios where criteria are interdependent or non-hierarchical. ANP extends the capabilities of AHP by accommodating feedback loops and interdependencies among criteria. It allows for a more comprehensive analysis of complex decision structures, making it suitable for systems with intricate relationships. But, introduces additional complexity, which can make it challenging to implement and interpret, especially for users unfamiliar with the method. It also requires extensive data collection and expert input to model relationships accurately [20,74]. SWARA offers a straightforward and systematic approach for determining criteria weights without the need for pairwise comparisons. It allows decision-makers to assess criteria based on their relative importance directly, simplifying the decision process. On the other hand, SWARA may oversimplify decision contexts by assuming that criteria are independent and equally significant. CoCoSo addresses the limitations of traditional MCDM methods by considering proportional assessments rather than pairwise comparisons. It offers a more flexible and intuitive approach to decision-making in dynamic and uncertain environments. However, CoCoSo's reliance on proportional assessments may require a thorough understanding of the decision context and criteria interactions. It may also be challenging to apply in situations with a large number of criteria or alternatives.

In the context of evaluating residential buildings for pandemic resilience within the sustainable development framework, the choice of SWARA and CoCoSo for this research is justified by their simplicity, transparency, and suitability for capturing the relative importance of criteria without relying heavily on pairwise comparisons. SWARA provides a straightforward method for determining criteria weights, which can be particularly beneficial when dealing with diverse stakeholders with varying levels of expertise. CoCoSo, on the other hand, offers a more flexible approach that accommodates proportional assessments and dynamic decision contexts, making it suitable for addressing the multifaceted challenges of pandemic resilience in residential buildings [21].

In conclusion, the overview of sustainable building assessment tools and decision-making approaches underscores the complexity inherent in evaluating building sustainability criteria, especially concerning pandemic resilience. Traditional methodologies like AHP and ANP, while valuable, may exhibit limitations in addressing uncertainties characteristic of pandemic environments. Nonetheless, the emergence of SWARA and CoCoSo as viable MCDM methods signifies their potential to encapsulate the multifaceted dimensions of sustainability, particularly amid global health crises like COVID-19. Their simplicity, transparency, and adaptability underscore their capacity to navigate the challenges of pandemic resilience within sustainable development frameworks. As we progress to the subsequent phase of inquiry, it is apparent that diverse perspectives and methodologies have enriched our comprehension and practices in sustainable building assessment and decision-making. Through ongoing exploration and refinement, resilient and adaptive building practices can be cultivated, fostering robust communities and environmental stewardship.

## 3 Research methodology

**Fig. 1:**
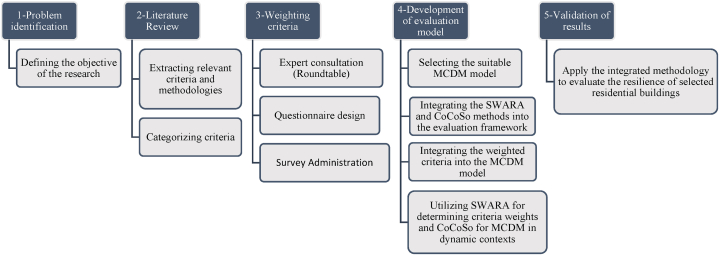
Step by step research methodology flowchart.

Building upon the insights gleaned from an extensive review of the background literature, which illuminated critical gaps and deficiencies in current approaches to evaluating residential building sustainability amidst pandemic conditions, this research presents a systematic methodology to assess the resilience of residential buildings in pandemics. By enhancing pandemic resilience, policymakers, architects, and builders can contribute to safer communities. The study aims to develop a framework for sustainable construction practices. The evaluation process is a Multi-Criteria Decision Making (MCDM) process that includes the characteristics of a building that maintain the health and comfort of residents during quarantine and prevent the spread of pathogens [19]. In the research paper, various methods have been suggested to evaluate criteria such as AHP, ANP, SWARA, etc. However, the SWARA method has been identified as the most appropriate and optimal method for the present research [47]. The conventional approaches, such as AHP, may struggle to accommodate the dynamic and uncertain nature of pandemic environments. To address this issue, the methodology proposed in this study takes a different approach. It uses a multi-faceted approach anchored in SWARA and CoCoSo methods. Both AHP and SWARA methods are capable of estimating the weight of criteria for solving MCDM problems with M number of options and N evaluation criteria. The SWARA method has been employed to determine the criteria weights in a robust framework, which allows decision makers to express judgments flexibly across a wider spectrum (ranging from 0 to 1) and minimize the need for exhaustive pair-wise comparisons. Subsequently, the CoCoSo method has been utilized, which integrates the Weighted Sum Model (WSM) and Weighted Product Model (WPM) approaches, to rank options based on multiple criteria [21,75]. These methods offer unparalleled adaptability and reliability in addressing the complex interplay of factors inherent in pandemic resilience assessment. By integrating these methodologies, the research aims to provide a comprehensive framework for evaluating residential building sustainability, with a specific focus on criteria conducive to pandemic resistance and public health. The methodology's stepwise process, encompassing criteria extraction, questionnaire design, criteria weighting, and multi-criteria decision-making, ensures a rigorous and systematic evaluation of residential building resilience under diverse pandemic scenarios. Furthermore, the method's flexibility enables stakeholders to account for evolving dynamics, such as technological advancements and shifting societal needs, thereby ensuring the continued relevance and applicability of the framework in an ever-changing landscape. In essence, the research methodology embodies a proactive and forward-thinking approach to sustainable construction, driven by the imperative of safeguarding public health and enhancing societal resilience in the face of unprecedented global challenges. It's important to note that this study was started at the beginning of the COVID-19 pandemic when global efforts were being made to understand the virus and find effective treatments. Initially, the research faced challenges in accessing resources dedicated to investigating sustainable strategies for residential buildings during quarantine. However, as knowledge and resources improved over time, the research methodology adapted to include the latest scientific insights and data. The step-by-step methodology is illustrated in Fig. 1.

The methodology of this research begins with problem identification, recognizing the urgent need to assess residential buildings' resilience to pandemics within sustainable construction frameworks, prompted by the global impact of the COVID-19 outbreak (see Table 1). Objectives are set to develop a comprehensive framework for this assessment. An extensive literature review follows, extracting relevant criteria and methodologies from previous studies. Criteria for evaluating sustainable buildings in pandemic conditions were extracted through a comprehensive review of literature, research, and previous studies. Given that, in times of unprecedented global health crises, such as the emergence of a novel pandemic, the imperative to prioritize the implementation of SDGs becomes increasingly pronounced. The SDGs offer a multifaceted framework capable of addressing pressing challenges, including water and food scarcity, while simultaneously enhancing public health and overall well-being. By steadfastly adhering to the principles outlined in the SDGs, societies can not only mitigate the immediate threats posed by pandemics but also harness these challenges as catalysts for transformative global change. Moreover, by fortifying collective resilience against genetically mutated viruses, the well-being of present and future generations can be safeguarded, thereby ensuring a brighter and more sustainable future for humankind [16]; according to the aforementioned considerations, in the next step, criteria that in addition to raising the level of building hygiene and protection of occupants against pandemic conditions, by observing the standards of green structures (such as the use of clean and renewable energy, optimal use of resources, water and energy, waste management, etc.) cause advancing SDGs; Included as the final elected criteria. A mixed sampling approach was adopted with a team of experts to derive pertinent and appropriate criteria from a wide range of options. This method combined purposive and snowball techniques to ensure a comprehensive representation of expertise spanning various fields relevant to sustainable building practices and pandemic resilience. The objective was to select 15 experts possessing specialized knowledge crucial to the research objectives from diverse backgrounds, including civil engineering, environmental science, architecture, project management, and molecular biology [[76], [77], [78]]. Through virtual roundtable discussions, these experts engaged in rigorous debates, exchanged valuable insights, and collectively arrived at a consensus regarding the final set of criteria. This collaborative endeavor aimed to capture a diverse range of perspectives, thereby enhancing the richness and breadth of the data gathered for the study. This set was identified for inclusion in the final questionnaire, as depicted in Table 2. Key criteria for evaluating resilience are identified and categorized into health and safety, resource management, and comfort groups. The final selected criteria in the form of a questionnaire were provided to experts. Survey respondents were chosen to represent a mixture of backgrounds including cell biology and molecular biology, architectural engineering, project management, environmental engineering, and genetics (with the academic rank of full professor and assistant professor) to ensure a comprehensive assessment of the criteria from various perspectives. This approach aimed to capture diverse viewpoints and insights that are essential for robust decision-making in evaluating pandemic-resistant sustainable residential buildings. The survey employed a purposive sampling method to select experts and professionals with relevant expertise and experience in the field of sustainable building practices and pandemic resilience. This approach ensured that respondents possessed the necessary knowledge and insights to provide informed opinions on the selected criteria. Experts in relevant fields assessed each criterion on a scale ranging from 1 to 10, where a score of 1 denoted the least importance and 10 indicated the highest significance. This standardized rating system enabled the quantification of experts' perceptions regarding the relative importance of each criterion in evaluating pandemic-resistant sustainable residential buildings. The survey instrument was disseminated electronically via a secure online platform, specifically Google Forms, enabling respondents to conveniently access and complete the questionnaire remotely. Given that the research was conducted during the peak of the COVID-19 pandemic, remote administration of the questionnaire proved to be the most effective and practical method, facilitating efficient data collection while mitigating logistical challenges inherent in traditional paper-based surveys. The average scores obtained from the expert panel were used to determine the relative importance of each criterion in the evaluation process, ensuring transparency and expert consensus in the criteria weighting process. The average score of each item obtained from the questionnaire, which was reviewed and answered by experts, can be seen in Table 2. After achieving the desired results of the questionnaire, they were used to weigh the criteria. Criteria weighting stage is one of the most important steps in MCDM problems. A MCDM model is selected and integrated with SWARA and CoCoSo methods.

**Table 1:** commonly assessed criteria, MCDM methods, and their application in reviewed literatures.

Author(s)	Application	Commonly Assessed Criteria	MCDM method
**Keshavarz et al.** [68]	evaluation of construction equipment with sustainability considerations	Socio-economic criteria- Engineering criteria- Environmental criteria	combination of the extended SWARA, fuzzy CRITIC and fuzzy EDAS methods.
**Hannan Amoozad Mahdiraji et al.** [69]	Prioritizing and Analysing Key Factors of Sustainable Architecture	environmental sustainability, energy consumption reduction, and green building practices.	BWM in conjunction with the COPRAS method.
**Ighravwea et al**. [70]	selecting the most suitable maintenance strategy for public buildings maintenance using sustainability criteria.	reducing energy emissions of CO2, building expenses, achieving the highest degree of thermal comfort	SWARA, WASPAS and ARAS
**Sherif Mahmouda et al.** [34]	to develop a sustainability assessment tool for existing buildings.	Site and ecology, Transportation, Energy and water efficiency, Material and waste, Indoor environmental quality, Building management	Fuzzy multi-criteria decision-making technique
**Abdulrahman Haruna et al.** [71]	comprehensive evaluation of construction, design, and management aspects to enhance sustainability	energy efficiency, greenhouse gas emissions, material usage, and environmental impacts	The method integrates BIM with the ANP
**Zavadskas et al.** [72]	Assessment of a Healthy and Safe Built Environment According to Sustainable Development Principles	Economic, social, environmental criteria included price and population density, the number of medical institutions and crime rate, green spaces and air pollution.	combination of the CILOS, IDOCRIW and COPRAS, SAW, TOPSIS, EDAS
**Al-Shammari et al.** [73]	to prioritize and rank factors related to rework in construction projects	cost, time, and quality of construction projects	a combined algorithm based on DEMATEL and ANP methods
**Natasha Khalil et al.** [49]	to evaluate the importance of different indicators and criteria related to building performance and user risks in Malaysian higher education buildings	functional performance, technical performance, indoor environmental performance, health risk, and safety risk.	AHP
**Rouzbeh Shad et al.** [54]	Developing an Iranian Green Building Assessment Tool (IGBT) for office buildings	Energy efficiency, water efficiency	AHP
**Hikmat H. Ali et al.** [55]	to develop an effective green building rating system for residential units in Jordan, considering local conditions and stakeholder input	Site selection, Energy efficiency, Water efficiency, Material and resources, Indoor environment quality, Waste and pollution, Economics,	AHP and development of the SABA Green Building Rating System
**Eglė Klumbytė et al**. [60]	sustainability assessment of municipal buildings, particularly focusing on the management of social housing and optimization of government and municipal facilities management	compliance regulations, economic ratio calculations, and meeting established criteria related to social housing management.	COPRAS, WASPAS
**Dobrovolskienė et al**. [61]	assessing the sustainability of a real estate project, comparing alternatives, and evaluating the level of value proposition against competitors.	CO2 emissions, Health and well-being, Innovative technologies employed, Use of renewable energy, Safety of infrastructure, Cost of technology	SAW
**Akhanova et al**. [66]	to weight the assessment items of a multi-criteria assessment framework for building sustainability	energy, water, site selection, indoor environmental quality, materials, and waste	SWARA
**Kamranfar et al.** [67]	Identifying and ranking barriers to green construction development	Economic, environmental, cultural, and social criteria	DEMATEL, ANP
**Mehdi Keshavarz Ghorabaee et al.** [68]	to evaluate construction equipment with sustainability considerations	environmental impact, energy efficiency, durability, cost-effectiveness, safety features	SWARA, CRITIC and Fuzzy EDAS method
**This study**	evaluating residential buildings in terms of resistance in pandemic conditions with a sustainable construction approach	Health and safety Comfort Resources management	a combined algorithm based on SWARA and COCOSO methods

**Table 2:** Selected criteria for evaluating residential buildings in pandemic conditions with a sustainable development approach [9,[23], [24], [25], [26], [27],^34^,^35^,^40^,^48^,^79^].

Number	Criteria code	Criteria	Average score obtained from the questionnaire	Category
1	C1	Sunlight (gained via orientation, openings, or open blinds).	8.62	Health and safety
2	C2	Using homogeneous materials with the least number of pores that do not create suitable conditions for the survival and residence of the virus.	7.14	Health and safety
3	C3	Regularly monitoring of wastewater to detect the possible presence of SARS-COVID-19 virus or other pathogens.	5.76	Resources management
4	C4	Disinfection of wastewater using UV radiation, chlorine dioxide or other suitable methods to combat COVID-19 virus or other dangerous viruses.	7.1	Resources management
5	C5	Using High-Efficiency Particulate Air filters (HEPA) capable of removing at least 99 % of particles of a specified size range.	7.38	Health and safety
6	C6	Providing a relative humidity of 40 %–60 % which improves air quality and reduces the persistence of the virus in aerosol particles.	7.5	Health and safety
7	C7	Provide a reliable power supply for all major building operations, health program management, and air conditioning. Preferably by creating local sources with low carbon consumption (renewable energy).	6.81	Resources management
8	C8	Considering specifically dedicated collecting, transporting, and waste management systems for quarantined sections or houses.	8	Health and safety
9	C9	Flexible and pliable entry design to adapt as a decontamination sector at the time of outbreaks.	8.05	Health and safety
10	C10	Provide additional room to quarantine infected people at the house, preferably with a separate bathroom.	7.95	Health and safety
11	C11	Considering landscapes with natural green space, exercise equipment and other amenities that improve the physical, and mental health of residents.	8.24	Health and safety
12	C12	Implementing roof gardens, green walls, balconies, or patios for the building, as far as possible.	7.67	Comfort
13	C13	View of greenery from windows.	8.43	Health and safety
14	C14	Flexibility of the interior spaces of the building with the possibility of changing the functions in special conditions.	6.75	Comfort
15	C15	Taking advantage of natural lighting and heat of solar radiation by the optimized orientation of the building	8.24	Resources management
16	C16	Applicating household water purification technologies.	7	Resources management
17	C17	Technical-plant engineering measures implementation like well-maintained plumbing, sealed bathroom drains, backflow valves on sprayers and faucets.	7.1	Health and safety
18	C18	Consideration of at least two bathrooms.	8	Health and safety
19	C19	Utilization of new and smart digital technologies for waste collection and separation.	7.1	Resources management
20	C20	Application of modern technologies in the field of control and monitoring of the elderly or sick.	8.14	Comfort
21	C21	Continuous air ventilation along with appropriate providing of indoor air quality indicators such as temperature and humidity, which is vital for the health and comfort of the residents.	8.05	Health and safety
22	C22	Use of intelligent systems and technologies to control energy consumption, which has been increased during the house quarantine period by occupants who are spending most of their time at the house and doing their work at the house.	6.95	Resources management
23	C23	Creating a standard social distance (at least 1 m) by assigning appropriate dimensions to building spaces such as corridors, stairs, lobbies, and entrance spaces.	7.55	Health and safety
24	C24	Design considerations for easy accommodation and movement of wheelchairs, people with canes, trolleys, stretchers, and beds in corridors, passageways, stairways, and other access routes.	7.38	Health and safety
25	C25	Decreasing the use of horizontal surfaces such as ledges or edges that cause the virus to reside and spread.	4.67	Health and safety
26	C26	Optimizing the layout of the building plan to improve the performance of natural ventilation and create a natural flow of air in the house.	8.24	Health and safety
27	C27	Setting the air quality monitoring system of PM10, PM2.5, and CO2 concentration, with storage of at least one year's worth of data.	6.14	Health and safety
28	C28	Installation of an online system for monitoring and evaluating water quality indicators, with the possibility of storing monitoring data that is available at any time needed for property management personnel.	6.19	Resources management
29	C29	Use of smart house technologies in the fields of House appliances control, house lighting and ventilation control, security and monitoring of environmental affairs, and work and life services or remote monitoring systems or systems connected to the smart city system, for the comprehensive management of people and vehicles in pandemic times.	7.71	Comfort
30	C30	Consideration of emergency evacuation passageways and corridors in the design process and providing all of the requirements related to emergency exit and evacuation and their permanent availability for emergencies.	7.33	Health and safety
31	C31	having design requirements for outdoor and indoor public spaces suitable for all ages. This includes designing internal and external passageways without obstacles, ensuring walls and columns along internal paths have no sharp corners, providing handles and safety railings for accessibility and safety and also allocating convenient space for elevators, and ensuring quick access to medical resources	7.67	Comfort
32	C32	Airtight performance of exterior windows.	7.62	Resources management
33	C33	Designing a regular program for cleaning and disinfection of water storage facilities such as drinking water tankers and reservoir facilities that must be cleaned and disinfected at least once every six months to minimize the risk of disease transmission from contaminated water.	7.33	Health and safety
34	C34	Providing spaces for growing plants that, especially during quarantine, cause the mental and physical health of residents and the health benefits of eating plants grown by residents themselves.	7.38	Health and safety

### 3.1 (SWARA) method

Weight calculation important levels of the SWARA method are as follows.

(1) Arranging the indicators: In the beginning, the indicators favored by the decision makers are chosen and arranged as the final indicators according to their importance. Accordingly, the most important indicators are in the higher categories and the less important indicators are in the lower categories.
(2) Assessing each index's relative importance (Sj): In this step, the relative importance of each index compared to the previous more important index must be determined. In this process in the SWARA method, this value is indicated by (Sj).
(3) Calculation of (Kj) coefficient: The (Kj) coefficient is calculated from Equation (1), which is a function of the relative importance of every index.

(1) Kj=Sj+1

(4) Calculating each index's initial weight: Equation (2) can calculate indices initial weight. But this must also be taken into account that the first index weight as the most important index is considered equal to 1.

(2) $$q_{j}=\frac{q_{j-1}}{K_{j}}$$

(5) Final normal weight Calculation: Calculating equation (3) is the final level of the SWARA method, it calculates indicators final weight also known as the Normalized Weight.

(3) $$w_{j}=\frac{q_{j}}{\sum q_{j}}$$

Now, by assigning weights appropriate to each criterion, the criteria can be used in the MCDM process. The method (MCDM) used in this research is the CoCoSo method. The CoCoSo method is one of the new MCDM techniques, which was presented by Yazdani et al., In 2018. In this method, a compromised combined solution for ranking options is presented. The steps of this method are given below [21].

### 3.2 (CoCoSo) method

#### 3.2.1 Decision matrix formation

In this part, m options are assessed using n criteria. Accordingly, each option is scored relying on each criterion. These scores can be based on quantitative and real values or qualitative and theoretical. In either case, a decision matrix m*n must be formed.

#### 3.2.2 Decision matrix normalization

In this part, using equations (4), (5) the decision matrix is normalized.

(4) $$r_{ij}=\frac{x_{ij}-\min\;x_{ij}}{\max\;x_{ij}-\min\;x_{ij}}\;For\;positive\;criteria$$

(5) $$r_{ij}=\frac{\max\;x_{ij}-x_{ij}}{\max\;x_{ij}-\min\;x_{ij}}\;For\;negative\;criteria$$

#### 3.2.3 Calculation of weight multiplication (S) and weight power (P)

In this step, using equations (6), (7), the weight multiplication (S) and weight power (P) are calculated, respectively. In fact, the value of (S) is equal to the sum of the multiplication of the criteria weight in the normal matrix for each option; And the value of (P) is equal to the sum of the values of the normal matrix to the power of the criteria weight.

(6) $$S_{i}=\sum_{j=1}^{n}\left(W_{j}r_{ij}\right)$$

(7) $$P_{i}=\sum_{j=1}^{n}{\left(r_{ij}\right)}^{W_{j}}$$

#### 3.2.4 Evaluate options based on three strategies

In this step, based on relationships (8), (9), and (10), the evaluation of options is based on three strategies. Equation (9) expresses the arithmetic mean of the values P and S. In equation (10), the sum of the best choices is in the S and P values. Equation (10) is a compromise between S and P. In this relation, λ is determined by the decision maker, but in the 0.5 mode, it has considerable flexibility.

(8) $$K_{ia}=\frac{P_{i}+S_{i}}{\sum_{i=1}^{m}\left(P_{i}+S_{i}\right)}$$

(9) $$K_{ib}=\frac{S_{i}}{\min\;S_{i}}+\frac{P_{i}}{\min\;P_{i}}$$

(10) $$K_{ic}=\frac{\lambda S_{i}+{\left(1-\lambda \right)P}_{i}}{\lambda \;\max\;S_{i}+\left(1-\lambda \right)\max\;P_{i}}$$

#### 3.2.5 Determine the final score of the options

In this step, based on equation (11), the final score of each option is calculated and the options are ranked based on it.

(11) $$k_{i}={\left(k_{ia}k_{ib}k_{ic}\right)}^{\frac{1}{3}}+\frac{1}{3}\left(k_{ia}+k_{ib}{+k}_{ic}\right)$$

After selecting ranking criteria, a final model for evaluating residential buildings against pandemics was created using the MCDM method of Swara-CoCoCo. The approach was aimed at achieving SDGs. In the next stage, three buildings in district 1 of Tehran city in Iran were included in the model and evaluated. The integrated methodology is applied to evaluate selected residential buildings' resilience to pandemics, interpreting results to identify strengths, weaknesses, and areas for improvement. The research concludes with recommendations for enhancing pandemic resilience in residential buildings within sustainable construction frameworks, documented for dissemination to stakeholders and the scientific community. The results of this research can be used to evaluate residential buildings in a given area. It can also identify buildings that do not meet the minimum approved standards and prepare them to confront pandemic conditions. Additionally, the final selected criteria can be used as a model for buildings that are still in the design stage.

## 4 Data analysis and results

Following the identification of appropriate methodologies and tools, the outcomes of this study serve as a foundational framework for evaluating sustainable residential buildings resistant to pandemic conditions. The evaluation process is guided by a comprehensive set of criteria derived from an extensive review of previous relevant research. Given the substantial number of criteria initially identified, a meticulous selection process ensued. Through expert consultation and consensus-building in a roundtable format, redundant or tangential criteria were pruned, yielding a refined set of pivotal criteria that comprehensively address the research objectives. Notably, from an initial pool of approximately 150 criteria, 34 were deemed pertinent and selected for inclusion in the final questionnaire. Distributed to a cohort of 250 identified specialists via email, the questionnaire requested respondents to assign scores to each research indicator on a scale ranging from 1 to 10, wherein 1 signified minimal importance and 10 denoted utmost significance. Subsequently, the average scores were tabulated, as presented in Table 2, based on responses from 42 participants. The attained response rate of 16.8 % was deemed satisfactory, given the predefined sample size and the specialized expertise of the respondents. Noteworthy was the substantial engagement observed among respondents specializing in sustainable building practices and pandemic resilience, underscoring the relevance and pertinence of the research. It is imperative to emphasize that the evaluating tool and its associated criteria elucidated in this study represent a general and comprehensive framework applicable to any residential building. While the criteria themselves remain constant, their prioritization, determined through the weighting process, may vary in response to contextual factors such as geographical location and temporal dynamics. This adaptability ensures the enduring relevance and applicability of the framework across diverse settings and evolving circumstances. In the subsequent phase, the results of the questionnaire, as delineated in Table 2, inform the weighting process of the criteria, leveraging the SWARA method to elucidate relative importance and guide decision-making processes effectively.

The process of calculating the weight for individual indicators is as follows: first, each of the 34 indicators that are arranged in descending order is compared with its previous index to determine how important it is from its previous index. Then, the weight of the indicators is calculated based on the SWARA method, which is given in Table 3.

**Table 3:** Results of SWARA method calculations.

Criteria code	Average score	Sj	Kj	qj	Wj
C1	8.6190	0	1	1	0.0744
C13	8.4286	0.1905	1.1905	0.84	0.0625
C11	8.2381	0.1905	1.1905	0.7056	0.0525
C15	8.2381	0	1	0.07056	0.0525
C26	8.2381	0	1	0.7056	0.0525
C20	8.1429	0.0952	1.0952	0.6442	0.0479
C9	8.0476	0.0952	1.0952	0.5882	0.0438
C21	8.0476	0	1	0.5882	0.0438
C8	8	0.0476	1.0476	0.5615	0.0418
C18	8	0	1	0.5615	0.0418
C10	7.9524	0.0476	1.0476	0.5360	0.0399
C29	7.7143	0.2381	1.2381	0.4329	0.0322
C12	7.6667	0.0476	1.0476	0.4132	0.0307
C31	7.6667	0	1	0.4132	0.0307
C32	7.6190	0.0476	1.0476	0.3944	0.0293
C23	7.55	0.0690	1.0690	0.3690	0.0275
C6	7.5	0.05	1.05	0.3514	0.0261
C5	7.3810	0.1190	1.1190	0.3114	0.0234
C24	7.3810	0	1	0.3140	0.0234
C34	7.3810	0	1	0.3140	0.0234
C30	7.3333	0.0476	1.0476	0.2997	0.0223
C33	7.3333	0	1	0.2997	0.0223
C2	7.1429	0.1905	1.1905	0.2518	0.0187
C4	7.0952	0.0476	1.0476	0.2403	0.0179
C17	7.0952	0	1	0.2403	0.0179
C19	7.0952	0	1	0.2403	0.0179
C16	7	0.0952	1.0952	0.2194	0.0163
C22	6.9524	0.0476	1.0476	0.2095	0.0156
C7	6.8095	0.1429	1.1429	0.1833	0.0136
C14	6.75	0.0595	1.0595	0.1730	0.0129
C28	6.1905	0.5595	1.5595	0.1109	0.0083
C27	6.1429	0.0476	1.0476	0.1059	0.0079
C3	5.7619	0.3810	1.3810	0.0767	0.0057
C25	4.6667	1.0952	2.0952	0.0366	0.0027

It is now possible to evaluate and compare sustainable residential buildings against pandemic conditions by having weighted criteria. In this study, the evaluation process involved a meticulous assessment of three buildings located in District 1 of Tehran, Iran. These buildings were strategically chosen to represent the affluent and well-developed nature of the district, characterized by superior standards and contemporary amenities such as rooftop gardens, green walls, smart home technologies, and state-of-the-art HVAC systems. Residential properties in this area were selected due to their alignment with these high standards and their incorporation of advanced features, making them ideal candidates for the study. The selection of District 1 for sampling was driven by its reputation as a prosperous and forward-thinking region within the city, boasting a plethora of modern and environmentally sustainable building options. These attributes not only provided a more diverse and sophisticated sample set but also presented a more rigorous and challenging evaluation scenario for our research model. Furthermore, the choice of buildings in District 1 allowed for a broader coverage of evaluation criteria within our proposed model. This enhanced coverage not only enriched our analysis but also provided a robust basis for academic discussion and comparison. In conclusion, the utilization of buildings from District 1 offered a compelling and representative sample for our study, facilitating a comprehensive assessment of structural integrity, environmental sustainability, and pandemic resilience. Moreover, it underscored the relevance and applicability of our evaluation model to various residential settings across the city. In the subsequent sections, these buildings will undergo evaluation and comparison using weighted criteria and the CoCoSo MCDM method. The chosen trio for assessment and comparison comprises residential apartments situated in District 1 of Tehran.

### 4.1 Formation of decision matrix

The first stage in this method is to set up a decision matrix. The decision matrix of this method consists of 34 research criteria and 3 buildings. The evaluation of 3 buildings is based on 34 criteria that are scored based on the range of 1–10 in the visits that have been made to them. The decision matrix is given in Table 4.

**Table 4:** The Decision CoCoSo matrix.

Criteria Code	C1	C2	C3	C4	C5	C6	C7	C8	C9	C10	C11	C12
Fatima building	6	5	1	1	2	1	1	5	6	8	6	5
Noghreh kar building	8	2	1	1	3	1	3	3	4	9	6	10
Sekke building	8	5	1	1	4	1	5	5	6	1	6	10
Criteria Code	**C13**	**C14**	**C15**	**C16**	**C17**	**C18**	**C19**	**C20**	**C21**	**C22**	**C23**	
Fatima building	5	5	1	5	10	3	1	5	1	6	6	
Noghreh kar building	6	8	1	6	10	3	1	6	1	5	5	
Sekke building	6	6	1	7	10	3	1	7	1	5	5	
Criteria Code	**C24**	**C25**	**C26**	**C27**	**C28**	**C29**	**C30**	**C31**	**C32**	**C33**	**C34**	
Fatima building	7	6	1	1	4	4	3	5	1	2	7	
Noghreh kar building	5	5	1	1	6	7	3	5	1	1	5	
Sekke building	7	6	1	1	7	6	3	5	1	1	7	

### 4.2 Decision matrix normalization

In this step, the decision matrix is normalized based on equations (4), (5), which is given in Table 5.

**Table 5:** The normalized decision CoCoSo matrix.

Criteria Code	C1	C2	C3	C4	C5	C6	C7	C8	C9	C10	C11	C12
Fatima building	0.000	1.000	0.000	0.000	0.000	0.000	0.000	1.000	1.000	0.875	0.000	0.000
Noghreh kar building	1.000	0.000	0.000	0.000	0.500	0.000	0.500	0.000	0.000	1.000	0.000	1.000
Sekke building	1.000	1.000	0.000	0.000	1.000	0.000	1.000	1.000	1.000	0.000	0.000	1.000
Criteria Code	**C13**	**C14**	**C15**	**C16**	**C17**	**C18**	**C19**	**C20**	**C21**	**C22**	**C23**	
Fatima building	0.000	0.000	0.000	0.000	0.000	0.000	0.000	0.000	0.000	1.000	1.000	
Noghreh kar building	1.000	1.000	0.000	0.500	0.000	0.000	0.000	0.500	0.000	0.000	0.000	
Sekke building	1.000	0.333	0.000	1.000	0.000	0.000	0.000	1.000	0.000	0.000	0.000	
Criteria Code	**C24**	**C25**	**C26**	**C27**	**C28**	**C29**	**C30**	**C31**	**C32**	**C33**	**C34**	
Fatima building	1.000	1.000	0.000	0.000	0.000	0.000	0.000	0.000	0.000	1.000	1.000	
Noghreh kar building	0.000	0.000	0.000	0.000	0.667	1.000	0.000	0.000	0.000	0.000	0.000	
Sekke building	1.000	1.000	0.000	0.000	1.000	0.667	0.000	0.000	0.000	0.000	1.000	

### 4.3 4.3calculation of weight multiplication (S) and weight power (P)

In this section, using equations (6), (7), the values of weight multiplication (S) and weight power (P) are calculated. To calculate (S), the weight of the criteria calculated by the SWARA method must be multiplied by the normal matrix, and then the sum of the rows must be obtained from the numbers of the matrix, and to calculate (P), the normal matrix numbers must be brought to the power of the criteria weight. Then the sum of the rows must be obtained from the numbers of the matrix. The results are given in Table 6.

**Table 6:** Score options based on strategies.

	S	P	Ka	Kb	Kc
Fatima building	0.269	8.995	0.245	2.000	0.567
Noghreh kar building	0.366	11.919	0.324	2.688	0.752
Sekke building	0.462	15.869	0.431	3.483	1.000

### 4.4 Evaluate options based on three strategies

In this step, based on relationships (8), (9), and (10), the evaluation score of the options is determined based on 3 strategies, the results of which are given in Table 6.

### 4.5 Final rating and ranking of options

In this step, based on equation (11), the final score of each option is calculated and ranked accordingly. The results are given in Table 7, according to the results, the Sekke building is ranked first, Noghreh Kar is ranked second and Fatima is ranked third (see Table 8).

**Table 7:** Score and final ranking of options.

Buildings' names	Ka	Rank
Fatima	1.589	3
Noghreh Kar	2.124	2
Sekke	2.783	1

**Table 8:** Criteria prioritization by weight for assessing residential buildings in pandemic conditions with a sustainable development perspective.

Number	Criteria code	Criteria	Average score obtained from the questionnaire	Category
1	C1	Sunlight (gained via orientation, openings, or open blinds).	8.62	Health and safety
13	C13	View of greenery from windows.	8.43	Health and safety
11	C11	Considering landscapes with natural green space, exercise equipment and other amenities that improve the physical, and mental health of residents.	8.24	Health and safety
15	C15	Taking advantage of natural lighting and heat of solar radiation by the optimized orientation of the building	8.24	Resources management
26	C26	Optimizing the layout of the building plan to improve the performance of natural ventilation and create a natural flow of air in the house.	8.24	Health and safety
20	C20	Application of modern technologies in the field of control and monitoring of the elderly or sick.	8.14	Comfort
9	C9	Flexible and pliable entry design to adapt as a decontamination sector at the time of outbreaks.	8.05	Health and safety
21	C21	Continuous air ventilation along with appropriate providing of indoor air quality indicators such as temperature and humidity, which is vital for the health and comfort of the residents.	8.05	Health and safety
8	C8	Considering specifically dedicated collecting, transporting, and waste management systems for quarantined sections or houses.	8	Health and safety
18	C18	Consideration of at least two bathrooms.	8	Health and safety
10	C10	Provide additional room to quarantine infected people at the house, preferably with a separate bathroom.	7.95	Health and safety
29	C29	Use of smart house technologies in the fields of House appliances control, house lighting and ventilation control, security and monitoring of environmental affairs, and work and life services or remote monitoring systems or systems connected to the smart city system, for the comprehensive management of people and vehicles in pandemic times.	7.71	Comfort
12	C12	Implementing roof gardens, green walls, balconies, or patios for the building, as far as possible.	7.67	Comfort
31	C31	having design requirements for outdoor and indoor public spaces suitable for all ages. This includes designing internal and external passageways without obstacles, ensuring walls and columns along internal paths have no sharp corners, providing handles and safety railings for accessibility and safety and also allocating convenient space for elevators, and ensuring quick access to medical resources	7.67	Comfort
32	C32	Airtight performance of exterior windows.	7.62	Resources management
23	C23	Creating a standard social distance (at least 1 m) by assigning appropriate dimensions to building spaces such as corridors, stairs, lobbies, and entrance spaces.	7.55	Health and safety
6	C6	Providing a relative humidity of 40 %–60 % which improves air quality and reduces the persistence of the virus in aerosol particles.	7.5	Health and safety
5	C5	Using High-Efficiency Particulate Air filters (HEPA) capable of removing at least 99 % of particles of a specified size range.	7.38	Health and safety
24	C24	Design considerations for easy accommodation and movement of wheelchairs, people with canes, trolleys, stretchers, and beds in corridors, passageways, stairways, and other access routes.	7.38	Health and safety
34	C34	Providing spaces for growing plants that, especially during quarantine, cause the mental and physical health of residents and the health benefits of eating plants grown by residents themselves.	7.38	Health and safety
30	C30	Consideration of emergency evacuation passageways and corridors in the design process and providing all of the requirements related to emergency exit and evacuation and their permanent availability for emergencies.	7.33	Health and safety
33	C33	Designing a regular program for cleaning and disinfection of water storage facilities such as drinking water tankers and reservoir facilities that must be cleaned and disinfected at least once every six months to minimize the risk of disease transmission from contaminated water.	7.33	Health and safety
2	C2	Using homogeneous materials with the least number of pores that do not create suitable conditions for the survival and residence of the virus.	7.14	Health and safety
4	C4	Disinfection of wastewater using UV radiation, chlorine dioxide or other suitable methods to combat COVID-19 virus or other dangerous viruses.	7.1	Resources management
17	C17	Technical-plant engineering measures implementation like well-maintained plumbing, sealed bathroom drains, backflow valves on sprayers and faucets.	7.1	Health and safety
19	C19	Utilization of new and smart digital technologies for waste collection and separation.	7.1	Resources management
16	C16	Applicating household water purification technologies.	7	Resources management
22	C22	Use of intelligent systems and technologies to control energy consumption, which has been increased during the house quarantine period by occupants who are spending most of their time at the house and doing their work at the house.	6.95	Resources management
7	C7	Provide a reliable power supply for all major building operations, health program management, and air conditioning. Preferably by creating local sources with low carbon consumption (renewable energy).	6.81	Resources management
14	C14	Flexibility of the interior spaces of the building with the possibility of changing the functions in special conditions.	6.75	Comfort
28	C28	Installation of an online system for monitoring and evaluating water quality indicators, with the possibility of storing monitoring data that is available at any time needed for property management personnel.	6.19	Resources management
27	C27	Setting the air quality monitoring system of PM10, PM2.5, and CO2 concentration, with storage of at least one year's worth of data.	6.14	Health and safety
3	C3	Regularly monitoring of wastewater to detect the possible presence of SARS-COVID-19 virus or other pathogens.	5.76	Resources management
25	C25	Decreasing the use of horizontal surfaces such as ledges or edges that cause the virus to reside and spread.	4.67	Health and safety

## 5 Discussion

One of the significant outcomes of this research is the compilation of selected criteria, meticulously ranked by experts from a multitude of criteria gathered from various sources. The chosen weighted criteria of sustainable buildings resilient against pandemic conditions hold applicability across diverse domains. They can serve to inform updates to existing sustainable building regulations or be integrated into specific regulations dedicated solely to addressing this pressing issue. Moreover, these results offer valuable insights for guiding the design and construction of new buildings or retrofitting existing ones with a sustainability-oriented and pandemic-resilient approach. The emphasis lies in employing criteria that, tailored to the building's conditions, optimize resource utilization (time, cost, and supplies) while effecting the most desirable changes. Furthermore, the ranked criteria extracted from the questionnaire form the basis of the elected MCDM model for evaluating residential buildings. This MCDM framework represents a generalizable approach, poised for potential application in future pandemics, thereby ensuring preparedness for unforeseen challenges. Importantly, the selected criteria utilized in the MCDM model are foundational and enduring, applicable across contexts. Their prioritization, contingent upon the weighing process, may evolve over time, accommodating shifts in location, temporal considerations, and advancements in technology pertinent to residential building hygiene. Thus, the framework remains adaptable, offering a robust mechanism for addressing pandemic conditions within the realm of sustainable construction. The top 10 criteria with the highest weights compared to other criteria for evaluating sustainable buildings resistant to pandemic conditions from the questionnaire are sunlight (gained via orientation, openings, or open blinds), view of greenery from windows, considering landscapes with natural green space, exercise equipment, and other amenities that improve the physical and mental health of residents, taking advantage of natural lighting and heat of solar radiation by the optimized orientation of the building, optimizing the layout of the building plan to improve the performance of natural ventilation and create a natural flow of air in the house, application of modern technologies in the field of control and monitoring of the elderly or sick, flexible and pliable entry design to adapt as a decontamination sector at the time of outbreaks, continuous air ventilation along with appropriate provision of indoor air quality indicators such as temperature and humidity, which is vital for the health and comfort of the residents, considering specifically dedicated collecting, transporting, and waste management systems for quarantined sections or houses, installation of more than one bathroom.

Achieving the highest score entails ensuring that the building receives ample sunlight by strategically positioning windows and shutters. The reason for this lies in the fact that natural sunlight can provide both heat and Ultraviolet-C (UVC) waves, which have been found to be highly effective in managing bacterial and viral infections. This, in turn, can greatly aid in curbing the spread of infections [79]. One of the key components for a sustainable pandemic-resistant residential building is the ability to enjoy a view of green spaces through the windows. This factor has a significant impact on the mental well-being of residents, particularly during quarantine conditions. By incorporating windows and shutters that are suitable for residential buildings, natural light and heat can be maximized while also promoting a healthy atmosphere. Furthermore, natural sunlight and ventilation can work together to eliminate pathogens, reduce energy consumption, and align with SDGs. Ultimately, providing a view of open and green spaces can help to maintain the mental health of residents, making it an essential aspect of a sustainable pandemic-resistant residential building that addresses disease control and ensures a comfortable and healthy living environment. The criteria that harness natural lighting and solar radiation through optimized building orientation entail strategically positioning the structure to maximize exposure to sunlight throughout the day. By doing so, architects and designers can exploit natural light to illuminate interior spaces, reducing the reliance on artificial lighting sources and minimizing energy consumption. Furthermore, the efficient layout of the building plan is pivotal for enhancing natural ventilation and fostering a continuous flow of fresh air within the premises. This entails meticulous consideration of factors such as room placement, window positioning, and airflow pathways to facilitate passive cooling and air circulation, thereby enhancing indoor air quality and thermal comfort. Additionally, the installation of multiple bathrooms within residential units contributes to improved hygiene and sanitation practices, particularly during quarantine periods. Moreover, the incorporation of dedicated waste management systems tailored for quarantined sections or houses is essential for containing and disposing of potentially infectious waste materials safely and efficiently. These criteria, identified as essential elements in sustainable residential building design, represent a holistic approach to pandemic resilience, addressing both environmental sustainability and public health considerations.

DHR Spennemann's research, as documented in the Journal of Green Building [25], provides valuable insights into the significance of these design criteria within the context of sustainable residential development. Through a comprehensive examination of building design principles and their implications for pandemic resilience, Spennemann underscores the critical role of proactive design strategies in mitigating the transmission of infectious diseases within residential settings. By emphasizing the integration of natural lighting, ventilation optimization, and tailored waste management systems, Spennemann advocates for a paradigm shift towards more resilient and sustainable housing solutions. These insights serve as a guiding framework for architects, urban planners, and policymakers seeking to enhance the pandemic preparedness of residential structures while promoting environmental stewardship and human well-being.

The research conducted by Galym Tokazhanov and Aidana Tleuken represents a pivotal contribution to the discourse surrounding pandemic-resilient sustainable building design. Their study meticulously delineates crucial aspects of virus containment and the preservation of residents' well-being within residential settings. Notably, Tokazhanov and Tleuken underscore the critical role of air conditioning standards and mental health measures, shedding light on indicators pivotal for preventing virus transmission and ensuring occupants' psychological welfare. In parallel, the current research endeavors to enrich this discourse by delving into premiere criteria, particularly emphasizing the provision of greenery views from windows and the optimization of building space to enhance natural ventilation—a critical aspect in bolstering indoor air quality.

**Fig. 2:**
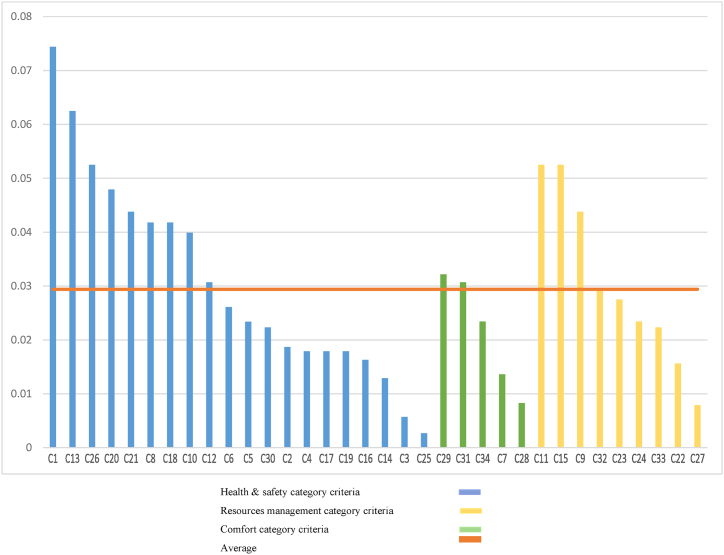
Criteria weight.

Diverging from Tokazhanov and Tleuken's focus on touchless technologies, this study, underpinned by the latest scientific insights into COVID-19 characteristics, accords greater weight to criteria addressing indoor air quality and airflow dynamics. Given the heightened risk of airborne transmission, criteria related to air conditioning systems and natural airflow assume paramount importance in this investigation, thus surpassing the significance of touchless technologies in the context of pandemic resilience strategies [80,81]. It is essential to take proactive measures during the initial design phase of residential buildings to effectively address pandemic resilience, which requires a paradigm shift in current design conventions. By categorizing criteria into distinct groups such as "health and safety, resource management, and comfort," a comprehensive evaluation framework emerges. Health and safety considerations are paramount in this framework, as can be seen in Fig. 2, which shows the criteria weights. It can be concluded that the health and safety category has the highest number of criteria with a weight higher than the total average weight. This observation resonates with Tokazhanov and Tleuken's framework [26], which similarly prioritizes health and safety criteria through the Dotmocracy technique, thus affirming their indispensable role in pandemic resilience strategies [64]. This research has identified a carefully selected set of key criteria through rigorous expert evaluation, which have the potential to inform future sustainable building regulations and construction practices. The top 10 criteria have been identified, which include optimized sunlight exposure, greenery views, and efficient natural ventilation systems, and present a holistic approach to pandemic resilience. These foundational elements exhibit adaptability to evolving contexts and technological advancements in residential building hygiene, ensuring the creation of sustainable, resilient living environments amidst emerging global challenges. In summary, this research is an important contribution to the scholarly discourse on pandemic-resilient sustainable building design. It aligns with insights from Tokazhanov and Tleuken's seminal study, emphasizing the critical imperative of prioritizing health and safety considerations in residential building design, particularly within the context of pandemics. This collective understanding underscores the necessity for proactive measures and informed decision-making to create sustainable, resilient living environments capable of withstanding the challenges of an ever-evolving global landscape.

In this comprehensive evaluation, the criterion focusing on "sunlight acquisition through strategic orientation, openings, or open blinds'" emerged as the preeminent factor, garnering the highest score across all assessed criteria. This criterion not only addresses the fundamental need for natural light within residential spaces but also underscores its critical role in promoting occupant well-being and mitigating health risks, particularly during pandemics.

Within the realm of comfort-oriented criteria, "utilizing intelligent service systems" stood out with the highest score. This criterion reflects the integration of advanced technologies to optimize comfort levels within residential environments, thereby enhancing overall livability and user experience. Moreover, the criteria emphasizing "landscape considerations incorporating natural green spaces, exercise amenities, and other facilities aimed at enhancing the physical and mental health of residents" alongside "maximizing natural lighting and harnessing solar radiation through optimized building orientations" achieved parity in scoring. This indicates their paramount importance in promoting sustainable resource management practices within residential developments. Such features not only contribute to ecological sustainability but also foster healthier and more resilient living environments, aligning closely with principles of sustainable development.

This nuanced understanding of criteria prioritization underscores the multidimensional nature of residential building evaluation, particularly within the context of pandemic resilience and sustainable development. By emphasizing factors such as access to natural light, integration of intelligent systems, and incorporation of green infrastructure, stakeholders can effectively enhance the quality, resilience, and sustainability of residential spaces in response to evolving societal needs and global challenges.

### 5.1 Multi-Criteria Decision Making model

Following the acquisition of desired results from the questionnaire, they can be utilized to assign weights to criteria, a principal step within MCDM problems. The process of evaluating pandemic-resistant sustainable residential buildings encompasses numerous influential indicators in the decision-making framework, delineating it as a discernible MCDM process [19]. In addition to the economic, social, and environmental facets of sustainable development, this study incorporates novel attributes of building characteristics that uphold resident health and comfort during quarantine while preventing pathogen transmission.

Presently, MCDM methods stand as the prevalent approach for assessing sustainable green buildings and prioritizing pertinent criteria and sub-criteria within environmental and construction science. The utilization of MCDM methods mandates the weighting and ranking of evaluation criteria based on their significance and impact. For instance, in the research conducted by Galym Tokazhanov and Aidana Tleuken [27] a scale of 0–5 was employed to evaluate and weigh indicators fostering epidemiological resilience in residential buildings through round table discussions with experts and stakeholders. Egl˙e Klumbyt˙e et al. employed COPRAS and WASPAS methods as MCDM models to evaluate sustainable urban buildings, offering a robust decision-making tool in municipal building management [60]. Similarly, Mohamed Abdel-Basset and Abduallah Gamal employed MCDM methods to evaluate and rank the criteria and dimensions of green buildings. They initially prioritized sub-criteria through the Delphi method and subsequently assessed the importance relativity of selected indicators using AHP [39]. Elaheh Yadegaridehkordi et al. investigated sustainability criteria for green buildings utilizing the GBI, a practical sustainability ranking tool in Malaysia [82]. They employed the DEMENTAL fuzzy method, an MCDM technique, to discern direct and indirect relationships between complex system criteria [19]. Abdulrahman Haruna utilized expert surveys to identify key factors influencing carbon emission reduction and energy consumption in sustainable buildings, employing the ANP within an MCDM model [71]. Hannan Amoozad Mahdiraji et al. combined the Best-Worst MCDM method and COPRAS to analyze sustainable architecture's key factors [69].

MCDM methods, including SWARA, AHP, ANP, and CoCoSo, are crucial for evaluating complex systems with conflicting criteria, particularly in assessing the sustainability of residential buildings amid pandemics. Each method presents strengths and weaknesses that shape its suitability for different research contexts. AHP offers a structured approach by decomposing problems into hierarchies, facilitating prioritization but may be subjective and struggle with complex scenarios. ANP extends AHP by accommodating feedback loops but may introduce complexity. SWARA simplifies criteria weight determination but may oversimplify decision contexts. CoCoSo addresses limitations by considering proportional assessments but requires a thorough understanding of criteria interactions. In evaluating residential buildings for pandemic resilience, SWARA, and CoCoSo are justified for their simplicity, transparency, and suitability in capturing criteria importance without heavy reliance on pairwise comparisons, thus addressing multifaceted challenges effectively [21]. The CoCoSo method is less susceptible to changes in criteria weight distribution or alterations in alternative options, offering enhanced reliability and consistency of results. Given its comprehensibility and utility, the CoCoSo method in conjunction with the SWARA method has been selected as the final MCDM method for this study. This decision is driven by the extensive number of criteria and the potential for evolving research outcomes to influence criteria importance [21,74,83].

### 5.2 Research limitations

The present research encountered some limitations that impeded the smooth conduct of the study. Specifically, the study's onset coincided with the beginning of the COVID-19 pandemic, presenting two significant challenges in preparing the research. Firstly, the novelty of the research topic contributed to the scarcity of available resources. While there were numerous sources and studies that investigated building sustainability criteria in general, only a limited number of sources had explored sustainable buildings that were resilient to pandemic conditions. This limitation complicated the gathering of relevant data and information necessary for the study.

Secondly, the outbreak of the COVID-19 pandemic in the early stages of the research presented a complex research environment. The unknown characteristics of the virus, lack of adequate information on methods to prevent its spread, and the absence of effective treatment methods for a considerable period posed significant challenges. Moreover, the focus of extensive global research on COVID-19 and the rapid updating of related information and discoveries necessitated the continuous update of available information to obtain valid and reliable results. The research team had to keep abreast of the latest developments in the field to ensure that the study's findings were relevant, accurate, and up-to-date.

The COVID-19 pandemic's impact on the study was also reflected in the changes in the research findings. For instance, in the initial stages of the pandemic, one of the primary concerns was the spread of the disease through contact with contaminated surfaces. However, the latest research indicates that this mode of transmission is not one of the primary and effective ways of transmission. Although the virus can survive on surfaces for several days, the efforts to culture the virus from these surfaces have been unsuccessful. Guidelines on infection control recommend that the highest risk of transmission of respiratory viruses is through infectious droplets caused by coughing, sneezing, and breathing [80,81]. The continuous updating of information about the COVID-19 pandemic also influenced the evaluation criteria used in the study. With the publication of new information, the importance and weight of criteria related to the use of touchless technologies were reduced, and the importance and weight of criteria related to ventilation and air filtration increased. Therefore, regular review of the World Health Organization website and other reliable up-to-date sources was crucial to access the latest information about the coronavirus [3]. In conclusion, the COVID-19 pandemic's impact on the study was significant, and the research team had to overcome several challenges to obtain valid and reliable results. The research team had to keep pace with the rapidly evolving updates about the pandemic to ensure that the study's findings were relevant and up-to-date. Despite the limitations, the research team was able to compile a comprehensive report that could inform future research in the field.

## 6 Conclusions

In this study, a comprehensive examination of the intricate relationship between pandemics and sustainable development within the realm of residential buildings has been undertaken. The emergence of COVID-19 has underscored the urgent need to address both challenges simultaneously, recognizing their profound implications for public health, societal well-being, and environmental sustainability. The pivotal role of residential structures as primary settings for safeguarding human health during pandemics, particularly evident amid widespread home quarantines aimed at curbing viral transmission, is asserted. This juncture necessitates a paradigm shift in the design, construction, and operation of residential buildings, aimed at enhancing their resilience to future pandemics while advancing broader sustainability objectives.

Through a rigorous methodology encompassing expert input and MCDM techniques, key criteria for evaluating the pandemic resilience of sustainable residential buildings have been identified and prioritized. By categorizing these criteria into domains of health and safety, resource management, and comfort, a nuanced understanding of the multifaceted considerations inherent in designing pandemic-resistant built environments is provided. The health and safety category had the highest number of criteria with a score greater than the total average. The experts found that "sunlight acquisition through strategic orientation, openings, or open blinds" was the most critical criterion, as it not only addresses the fundamental need for natural light but also plays a crucial role in promoting occupant well-being and mitigating health risks, particularly during pandemics. In terms of comfort-oriented criteria, the experts found that "utilizing intelligent service systems" was the most important factor. This criterion reflects the integration of advanced technologies to optimize comfort levels within residential environments, thereby enhancing overall livability and user experience. The experts also emphasized the importance of "landscape considerations incorporating natural green spaces, exercise amenities, and other facilities aimed at enhancing the physical and mental health of residents" and "maximizing natural lighting and harnessing solar radiation through optimized building orientations" in promoting sustainable resource management practices within residential developments. The results of the conducted questionnaire offer valuable insights into sustainable buildings that are capable of resisting pandemic conditions, an aspect that is applicable across various fields. These findings can inform the revision of current sustainable building regulations or the development of specific regulations aimed at enhancing pandemic resilience. Moreover, they serve as a reference for designing and constructing new buildings or retrofitting existing ones with sustainability and pandemic resistance in mind, thereby optimizing resource allocation and achieving desirable changes.

Furthermore, the integration of the SWARA-COCOSO MCDM method represents a methodological innovation in evaluating building resilience, offering a systematic and robust framework for decision-making amidst complex and uncertain contexts. By harnessing the strengths of both methodologies, decision-makers are provided with a comprehensive toolset for assessing and prioritizing pandemic resilience criteria, thereby facilitating informed and evidence-based interventions in building design and policy formulation.

Looking ahead, the operationalization of research findings into tangible policy measures and industry practices is advocated for. Specifically, the incorporation of identified criteria into green building regulations, accompanied by standardized assessment protocols to ensure consistency and accountability across residential construction projects, is recommended. Moreover, future research endeavors are encouraged to explore alternative MCDM methodologies and consider the evolving landscape of infectious diseases to enhance the adaptability and robustness of pandemic resilience frameworks.

In conclusion, this study represents a significant contribution to the burgeoning discourse on pandemic resilience and sustainable development in residential buildings. By elucidating the synergies between these critical domains and offering actionable insights for their integration, the aim is to catalyze transformative change in the built environment toward a more resilient, equitable, and sustainable future for all.

## Ethical Statement

This research did not involve human or animal experiments; therefore, ethical approval was not required.

## Clinical trial

This research did not involve a clinical trial.

## Data availability statement

The data supporting the findings of this study are available within the article and its supplementary materials. Additional data are available upon request from the corresponding author.

## Funding

This research did not receive any specific funding.

## CRediT authorship contribution statement

**Ali Heydari:** Writing – review & editing, Writing – original draft, Visualization, Validation, Supervision, Software, Resources, Project administration, Methodology, Investigation, Formal analysis, Data curation, Conceptualization. **Hamidreza Abbasianjahromi:** Writing – review & editing, Visualization, Validation, Supervision, Resources, Project administration, Methodology, Investigation, Conceptualization.

## Declaration of competing interest

The authors declare that they have no known competing financial interests or personal relationships that could have appeared to influence the work reported in this paper.
