# Supplementary material for: Evaluating the resilience of residential buildings during a pandemic with a sustainable construction approach
Source: Heliyon. 2024 May 14;10(10):e31006. doi: 10.1016/j.heliyon.2024.e31006 (PMC11128871; doi:10.1016/j.heliyon.2024.e31006)
Supplement: Multimedia component 1 [file mmc1.pdf]

Scoring the evaluation criteria of sustainable houses resistant to the pandemic situation

Research questionnaire

The covid-19 pandemic has forced many people to self-quarantine in their homes for health and safety reasons.So inadvertently, our homes were tested for flexibility and adaptability,

It is for this reason that we should adapt to such undesirable and unpredictable changes

(due to the emergence of possible epidemics in the future and the risk of the emergence of new unknown diseases, microbial terrorist attacks, viral mutations, etc.)

as soon as possible by improving the health and safety standards of homes.

Therefore, our future homes must respond to these needs in the event of possible future pandemics:

1)

How to minimize disease transmission

2)

How to minimize negative environmental effects

3)

How to improve the quality of life of people who spend most of their time at home during quarantine

In this research, in order to achieve a model for assessing houses in pandemic conditions, in addition to examining the criteria related to the health and well-being of residents during quarantine, the criteria of sustainable construction and green houses have also been considered.

Therefore, in the first step by studying previous researches and using the opinion of experts, a set of the most important building evaluation criteria to protect the health of the residents during the epidemic with a sustainable development approach has been collected in the form of a questionnaire, and then by Scoring the criteria and using them in a multi-criteria decision-making model, the buildings will be evaluated in terms of maintaining the health of the residents during the pandemic situation.

Please, according to the explanations provided, rate each of the following criteria, which are used to evaluate the level of resistance of the building against pandemic conditions and epidemic diseases, according to its importance and effectiveness with numbers from 1 to 10.

* Required

1. Email *

2. Sunlight (gained via orientation, openings or open blinds).

Mark only one oval.

- 1
- 2
- 3
- 4
- 5
- 6
- 7
- 8
- 9
- 10

3. Using homogeneous materials with the least amount of pores that do not create suitable conditions for the survival and residence of the virus.

Mark only one oval.

- 1
- 2
- 3
- 4
- 5
- 6
- 7
- 8
- 9
- 10

4.

Regular monitoring of wastewater to detect the possible presence of SARS-covid-19 virus or other pathogens.

Mark only one oval.

- 1
- 2
- 3
- 4
- 5
- 6
- 7
- 8
- 9
- 10

5. Disinfection of wastewater using UV radiation, chlorine dioxide or other suitable methods to combat Covid-19 virus or other dangerous viruses.

Mark only one oval.

- 1
- 2
- 3
- 4
- 5
- 6
- 7
- 8
- 9
- 10

6.

Using High-Efficiency Particulate Air filters (HEPA) capable of removing at least 99% of particles of a specified size range.

Mark only one oval.

- 1
- 2
- 3
- 4
- 5
- 6
- 7
- 8
- 9
- 10

7. Providing a relative humidity of 40% to 60% which improves air quality and reduces the persistence of the virus in aerosol particles.

Mark only one oval.

- 1
- 2
- 3
- 4
- 5
- 6
- 7
- 8
- 9
- 10

8. Provide a reliable power supply for all major building operations, health program management and air conditioning. Preferably by creating local sources with low carbon consumption (renewable energy).

Mark only one oval.

- 1
- 2
- 3
- 4
- 5
- 6
- 7
- 8
- 9
- 10

9. Considering specifically dedicated collecting, transporting and waste management systems for quarantined sections or houses.

Mark only one oval.

- 1
- 2
- 3
- 4
- 5
- 6
- 7
- 8
- 9
- 10

10. Flexible and pliable entry design to adapt as a decontamination sector at the time of outbreaks.

Mark only one oval.

- 1
- 2
- 3
- 4
- 5
- 6
- 7
- 8
- 9
- 10

11. Provide additional room to quarantine infected people at home, preferably with a separate bathroom.

Mark only one oval.

- 1
- 2
- 3
- 4
- 5
- 6
- 7
- 8
- 9
- 10

12. Considering landscapes with natural green space, exercise equipment and other amenities that improve the physical and mental health of residents.

Mark only one oval.

- 1
- 2
- 3
- 4
- 5
- 6
- 7
- 8
- 9
- 10

13. Implementing roof gardens, green walls, balconies or patios for the building, as far as possible.

Mark only one oval.

- 1
- 2
- 3
- 4
- 5
- 6
- 7
- 8
- 9
- 10

14. View of greenery from windows.

Mark only one oval.

- 1
- 2
- 3
- 4
- 5
- 6
- 7
- 8
- 9
- 10

15.

Flexibility of the interior spaces of the building with the possibility of changing the functions in special conditions.

Mark only one oval.

- 1
- 2
- 3
- 4
- 5
- 6
- 7
- 8
- 9
- 10

16.

Take advantage of natural lighting and heat of solar radiation by optimized orientation of the building.

Mark only one oval.

- 1
- 2
- 3
- 4
- 5
- 6
- 7
- 8
- 9
- 10

17. Application of household water purification technologies.

Mark only one oval.

- 1
- 2
- 3
- 4
- 5
- 6
- 7
- 8
- 9
- 10

18. Technical-plant engineering measures implementation like well-maintained plumbing, sealed bathroom drains, back flow valves on sprayers and faucets.

Mark only one oval.

- 1
- 2
- 3
- 4
- 5
- 6
- 7
- 8
- 9
- 10

19.

Consideration of at least two bathrooms.

Mark only one oval.

- 1
- 2
- 3
- 4
- 5
- 6
- 7
- 8
- 9
- 10

20.

Utilization of new and smart digital technologies for waste collection and separation.

Mark only one oval.

- 1
- 2
- 3
- 4
- 5
- 6
- 7
- 8
- 9
- 10

21. Application of modern technologies in the field of control and monitoring of the elderly or sick.

Mark only one oval.

- 1
- 2
- 3
- 4
- 5
- 6
- 7
- 8
- 9
- 10

22. Continuous air ventilation along with appropriate providing of indoor air quality indicators such as temperature and humidity, which is vital for the health and comfort of the residents.

Mark only one oval.

- 1
- 2
- 3
- 4
- 5
- 6
- 7
- 8
- 9
- 10

23. Use of intelligent systems and technologies to control energy consumption, which has been increased during the home quarantine period by occupants who are spending most of time at home and doing their work at home.

Mark only one oval.

- 1
- 2
- 3
- 4
- 5
- 6
- 7
- 8
- 9
- 10

24. Creating a standard social distance (at least 1 meter) by assigning appropriate dimensions to building spaces such as corridors, stairs, lobbies and entrance spaces.

Mark only one oval.

- 1
- 2
- 3
- 4
- 5
- 6
- 7
- 8
- 9
- 10

25. Design considerations for easy accommodation and movement of wheelchairs, people with canes, trolleys, stretchers and beds in corridors, passageways, stairways and other access routes.

Mark only one oval.

- 1
- 2
- 3
- 4
- 5
- 6
- 7
- 8
- 9
- 10

26. Decreasing the use of horizontal surfaces such as ledges or edges that cause the virus to residence and spread.

Mark only one oval.

- 1
- 2
- 3
- 4
- 5
- 6
- 7
- 8
- 9
- 10

27. Optimize the layout of the building plan to improve the performance of natural ventilation and create a natural flow of air in the house.

Mark only one oval.

- 1
- 2
- 3
- 4
- 5
- 6
- 7
- 8
- 9
- 10

28.

Set the air quality monitoring system of PM10, PM2.5, CO_2 concentration, with the possibility of at least one year storage of monitoring data.

Mark only one oval.

- 1
- 2
- 3
- 4
- 5
- 6
- 7
- 8
- 9
- 10

29. Installing an online system for monitoring and evaluating water quality indicators with the possibility of storage of monitoring data that is available any time that is needed for property management personnel.

Mark only one oval.

- 1
- 2
- 3
- 4
- 5
- 6
- 7
- 8
- 9
- 10

30. Use of smart home technologies in the fields of: Home appliances control, home lighting and ventilation control, security and monitoring of environmental affairs and work and life services or remote monitoring systems or systems connected to the smart city system, for the comprehensive management of people and vehicles in pandemic times.

Mark only one oval.

- 1
- 2
- 3
- 4
- 5
- 6
- 7
- 8
- 9
- 10

31. Emergency evacuation passageways and corridors must be considered in the design and provide all the requirements related to emergency exit and evacuation and always shall be available for emergency situations.

Mark only one oval.

- 1
- 2
- 3
- 4
- 5
- 6
- 7
- 8
- 9
- 10

32.

Outdoor and indoor public spaces have design requirements for all ages, such as the design of internal and external passageways without obstacles, walls and columns of internal paths without sharp corners considering the handles and safety railings, allocation convenient space for elevators and quick access to medical resources.

Mark only one oval.

- 1
- 2
- 3
- 4
- 5
- 6
- 7
- 8
- 9
- 10

33.

Airtight performance of exterior windows.

Mark only one oval.

- 1
- 2
- 3
- 4
- 5
- 6
- 7
- 8
- 9
- 10

34. Design a regular program for cleaning and disinfection of water storage facilities such as drinking water tankers and reservoirs facilities that must be cleaned and disinfected at least once every six months to minimize the risk of disease transmission from contaminated water.

Mark only one oval.

- 1
- 2
- 3
- 4
- 5
- 6
- 7
- 8
- 9
- 10

35. Providing spaces for growing plants that, especially during quarantine, cause mental and physical health of residents and the health benefits of eating plants grown by residents themselves.

Mark only one oval.

- 1
- 2
- 3
- 4
- 5
- 6
- 7
- 8
- 9
- 10

36. Sunlight (gained via orientation, openings or open blinds).

Mark only one oval.

- 1
- 2
- 3
- 4
- 5
- 6
- 7
- 8
- 9
- 10

37. Using homogeneous materials with the least amount of pores that do not create suitable conditions for the survival and residence of the virus.

Mark only one oval.

- 1
- 2
- 3
- 4
- 5
- 6
- 7
- 8
- 9
- 10

38.

Regular monitoring of wastewater to detect the possible presence of SARS-covid-19 virus or other pathogens.

Mark only one oval.

- 1
- 2
- 3
- 4
- 5
- 6
- 7
- 8
- 9
- 10

39. Disinfection of wastewater using UV radiation, chlorine dioxide or other suitable methods to combat Covid-19 virus or other dangerous viruses.

Mark only one oval.

- 1
- 2
- 3
- 4
- 5
- 6
- 7
- 8
- 9
- 10

40.

Using High-Efficiency Particulate Air filters (HEPA) capable of removing at least 99% of particles of a specified size range.

Mark only one oval.

- 1
- 2
- 3
- 4
- 5
- 6
- 7
- 8
- 9
- 10

41. Providing a relative humidity of 40% to 60% which improves air quality and reduces the persistence of the virus in aerosol particles.

Mark only one oval.

- 1
- 2
- 3
- 4
- 5
- 6
- 7
- 8
- 9
- 10

42. Provide a reliable power supply for all major building operations, health program management and air conditioning. Preferably by creating local sources with low carbon consumption (renewable energy).

Mark only one oval.

- 1
- 2
- 3
- 4
- 5
- 6
- 7
- 8
- 9
- 10

43. Considering specifically dedicated collecting, transporting and waste management systems for quarantined sections or houses.

Mark only one oval.

- 1
- 2
- 3
- 4
- 5
- 6
- 7
- 8
- 9
- 10

44. Flexible and pliable entry design to adapt as a decontamination sector at the time of outbreaks.

Mark only one oval.

- 1
- 2
- 3
- 4
- 5
- 6
- 7
- 8
- 9
- 10

45. Provide additional room to quarantine infected people at home, preferably with a separate bathroom.

Mark only one oval.

- 1
- 2
- 3
- 4
- 5
- 6
- 7
- 8
- 9
- 10

46. Considering landscapes with natural green space, exercise equipment and other amenities that improve the physical and mental health of residents.

Mark only one oval.

- 1
- 2
- 3
- 4
- 5
- 6
- 7
- 8
- 9
- 10

47. Implementing roof gardens, green walls, balconies or patios for the building, as far as possible.

Mark only one oval.

- 1
- 2
- 3
- 4
- 5
- 6
- 7
- 8
- 9
- 10

48. View of greenery from windows.

Mark only one oval.

- 1
- 2
- 3
- 4
- 5
- 6
- 7
- 8
- 9
- 10

49.

Flexibility of the interior spaces of the building with the possibility of changing the functions in special conditions.

Mark only one oval.

- 1
- 2
- 3
- 4
- 5
- 6
- 7
- 8
- 9
- 10

50.

Take advantage of natural lighting and heat of solar radiation by optimized orientation of the building.

Mark only one oval.

- 1
- 2
- 3
- 4
- 5
- 6
- 7
- 8
- 9
- 10

51. Application of household water purification technologies.

Mark only one oval.

- 1
- 2
- 3
- 4
- 5
- 6
- 7
- 8
- 9
- 10

52. Technical-plant engineering measures implementation like well-maintained plumbing, sealed bathroom drains, back flow valves on sprayers and faucets.

Mark only one oval.

- 1
- 2
- 3
- 4
- 5
- 6
- 7
- 8
- 9
- 10

53.

Consideration of at least two bathrooms.

Mark only one oval.

- 1
- 2
- 3
- 4
- 5
- 6
- 7
- 8
- 9
- 10

54.

Utilization of new and smart digital technologies for waste collection and separation.

Mark only one oval.

- 1
- 2
- 3
- 4
- 5
- 6
- 7
- 8
- 9
- 10

55. Application of modern technologies in the field of control and monitoring of the elderly or sick.

Mark only one oval.

- 1
- 2
- 3
- 4
- 5
- 6
- 7
- 8
- 9
- 10

56. Continuous air ventilation along with appropriate providing of indoor air quality indicators such as temperature and humidity, which is vital for the health and comfort of the residents.

Mark only one oval.

- 1
- 2
- 3
- 4
- 5
- 6
- 7
- 8
- 9
- 10

57. Use of intelligent systems and technologies to control energy consumption, which has been increased during the home quarantine period by occupants who are spending most of time at home and doing their work at home.

Mark only one oval.

- 1
- 2
- 3
- 4
- 5
- 6
- 7
- 8
- 9
- 10

58. Creating a standard social distance (at least 1 meter) by assigning appropriate dimensions to building spaces such as corridors, stairs, lobbies and entrance spaces.

Mark only one oval.

- 1
- 2
- 3
- 4
- 5
- 6
- 7
- 8
- 9
- 10

59. Design considerations for easy accommodation and movement of wheelchairs, people with canes, trolleys, stretchers and beds in corridors, passageways, stairways and other access routes.

Mark only one oval.

- 1
- 2
- 3
- 4
- 5
- 6
- 7
- 8
- 9
- 10

60. Decreasing the use of horizontal surfaces such as ledges or edges that cause the virus to residence and spread.

Mark only one oval.

- 1
- 2
- 3
- 4
- 5
- 6
- 7
- 8
- 9
- 10

61. Optimize the layout of the building plan to improve the performance of natural ventilation and create a natural flow of air in the house.

Mark only one oval.

- 1
- 2
- 3
- 4
- 5
- 6
- 7
- 8
- 9
- 10

62.

Set the air quality monitoring system of PM10, PM2.5, CO2 concentration, with the possibility of at least one year storage of monitoring data.

Mark only one oval.

- 1
- 2
- 3
- 4
- 5
- 6
- 7
- 8
- 9
- 10

63. Installing an online system for monitoring and evaluating water quality indicators with the possibility of storage of monitoring data that is available any time that is needed for property management personnel.

Mark only one oval.

- 1
- 2
- 3
- 4
- 5
- 6
- 7
- 8
- 9
- 10

64. Use of smart home technologies in the fields of: Home appliances control, home lighting and ventilation control, security and monitoring of environmental affairs and work and life services or remote monitoring systems or systems connected to the smart city system, for the comprehensive management of people and vehicles in pandemic times.

Mark only one oval.

- 1
- 2
- 3
- 4
- 5
- 6
- 7
- 8
- 9
- 10

65. Emergency evacuation passageways and corridors must be considered in the design and provide all the requirements related to emergency exit and evacuation and always shall be available for emergency situations.

Mark only one oval.

- 1
- 2
- 3
- 4
- 5
- 6
- 7
- 8
- 9
- 10

66.

Outdoor and indoor public spaces have design requirements for all ages, such as the design of internal and external passageways without obstacles, walls and columns of internal paths without sharp corners considering the handles and safety railings, allocation convenient space for elevators and quick access to medical resources.

Mark only one oval.

- 1
- 2
- 3
- 4
- 5
- 6
- 7
- 8
- 9
- 10

67.

Airtight performance of exterior windows.

Mark only one oval.

- 1
- 2
- 3
- 4
- 5
- 6
- 7
- 8
- 9
- 10

68. Design a regular program for cleaning and disinfection of water storage facilities such as drinking water tankers and reservoirs facilities that must be cleaned and disinfected at least once every six months to minimize the risk of disease transmission from contaminated water.

Mark only one oval.

- 1
- 2
- 3
- 4
- 5
- 6
- 7
- 8
- 9
- 10

69. Providing spaces for growing plants that, especially during quarantine, cause mental and physical health of residents and the health benefits of eating plants grown by residents themselves.

Mark only one oval.

- 1
- 2
- 3
- 4
- 5
- 6
- 7
- 8
- 9
- 10

This content is neither created nor endorsed by Google.

Google Forms
